# Supplementary material for: Patients’ preferences in dental care: A discrete-choice experiment and an analysis of willingness-to-pay
Source: PLoS One. 2023 Feb 27;18(2):e0280441. doi: 10.1371/journal.pone.0280441 (PMC9970100; doi:10.1371/journal.pone.0280441)
Supplement: S1 File — (PDF) [file pone.0280441.s001.pdf]

Why were you selected and where did we get your data from?

Your address was randomly selected for this study by your residents' registration office and will be used only once by us as part of this research project. A sample of people aged 30 and over in Berlin and Brandenburg will be surveyed. Your data set comes from the residents' registration office where you are registered. According to § 34 of the Federal Registration Act, residents' registration office data may be transmitted by residents' registration offices to other public bodies within the meaning of § 2(1)–(3)&(4) Sentence 2 of the Federal Data Protection Act in Germany and processed for research purposes in the public interest according to Article 5(1)b of the General Data Protection Regulation. You can disagree to the use of your data for this purpose at any time within the meaning of the Federal Data Protection Act at the address or e-mail address of your residents' registration office.

Questionnaire:

What role do selected treatment attributes play in the choice of a dental crown?

We are very pleased that you have taken the time to complete our questionnaire!

Your answers will of course be treated as **strictly confidential** and will be **used exclusively** for the purposes of the study and stored in a way that is inaccessible to third parties. The survey is **anonymous**, so that no conclusions can be drawn about your person in any way. Further information on data security can be found in the participant information on p. 3 and 4 of the questionnaire.

Your participation is voluntary. We understand if you do not wish to answer a question. If you find that you do not want to or cannot answer many questions, we ask you to refrain from participating.

As a thank you, you can take part in **a lottery of 20 shopping vouchers of 50 € value each**. You will find further information on the lottery in the lottery form in the envelope.

Please separate the first sheet with your address from the questionnaire. Please fold the completed questionnaire in half and return it to us in the enclosed envelope by dropping it in a Deutsche Post mailbox. The postage will be paid by the Technische Universität Berlin.

Please start with the participation declaration on the next page.

We thank you very much for your support!

Please start with the declaration of participation.

Declaration of participation

First, we need a confirmation from you that you agree to participate in the study. Please tick a box by placing a cross!

☐ Yes. I agree to participate in the study.

☐ No. I **do not** agree to participate in the study.

Further information about the research project and the study can be found in the participant information on p. 3 and 4 of the questionnaire. It is not possible to draw any conclusions about your person. Your participation is voluntary.
You can have the answers from your questionnaire deleted at any time. The entire questionnaire will then be deleted. In this case, please make a note of the questionnaire number (lower questionnaire margin, left-hand side) and contact us at 030/314 28701, or send us a written revocation by post. You find our address on the cover letter.

Note on completing the questionnaire:

You can answer the questionnaire on the following pages by placing a **cross** in the **appropriate box** below or next to your answer (see below) or, if necessary, by handwriting your answer.

☒ RIGHT: place a cross in the middle of the box

FALSE: unfortunately, a dash cannot be considered

■ CORRECTION: color the box and re-set the cross

Version: ST01

Participant information

What is the background to the research project and the study?

In the research project **MeDIoRI: Medical Devices: Incentives and Effects of Regulatory Instruments**, we have set ourselves the goal of investigating the legal framework of the German health care system. In doing so, we want to find out to what extent the requirements of the legislator (e.g., level of reimbursement by health insurance fund) have effects on patients and physicians, i.e., why, for example, a patient decides in preference for or against a medical treatment. In our considerations, we focus on medical devices, as little or no research has been done in this field so far. A dental crown is also a medical device. In the study on the present questionnaire, we examine the dental field because patients often must pay high out-of-pocket payments, especially for dental prostheses. Other attributes of a treatment may also influence the choice for or against it. The focus is on treatments at the dentist and here on the most common type of treatment of dentures in Germany: the **full crown** of a single tooth. In the questionnaire we use the term **dental crown**. You can find more information about the project at www.berlinhecor.de.

What happens to the questionnaire?

You can return the completed questionnaire to us in the enclosed return envelope by dropping it in a Deutsche Post mailbox. Our address is already printed on the return envelope. For data protection reasons, please do not add a return address on the envelope. The survey is anonymous. The postage for the return is paid by the Technische Universität Berlin. The returned questionnaire will be recorded electronically by us. The paper version of the questionnaire will be archived by us until the end of the project and then destroyed. The electronic version is stored on data carriers on site for a period of 10 years from the end of the project and then deleted.

Is your data secure?

In carrying out the study, great importance is attached to compliance with data protection regulations. Your answers will of course be treated as strictly confidential and will be used exclusively for the purposes of the study and stored on data carriers on site, inaccessible to third parties. The survey is anonymous, so that it is not possible to draw any conclusions about your person. For this purpose, we ask you to return only the completed questionnaire and the completed raffle form, sealed in the separate small envelope. Please only put the separate small envelope and the questionnaire into the large return envelope. Except for the raffle form, no documents may contain your name, address, or other personal information, such as your telephone number. When we receive your documents in the post, the questionnaire and the raffle form envelope will be immediately separated so that no link can subsequently be made between the two documents. The raffle forms of participants who have not won will be destroyed immediately after the shopping vouchers have been sent to the winners. The raffle forms of the winners are archived with us until the end of the project and then destroyed. For administrative reasons, the winners' contact details will be stored on data carriers on site for a period of 10 years from the end of the project and then deleted. The evaluation of your answers from the questionnaire is pseudonymized, i.e., we only know your questionnaire number. It is not possible to draw any conclusions about your person. We ask you not to leave any notes on the questionnaire or on any other documents returned to us that could lead to conclusions about your person. Should this nevertheless be the case, these notes will be blacked out immediately upon receipt by a member of staff who is not involved in the evaluation.

Can you have your data deleted?

Yes, you can have the answers deleted from your questionnaire at any time. The entire questionnaire will then be deleted. In this case, please make a note of the questionnaire number (bottom edge of the questionnaire) and contact us at 030/314 28701 or send us a written revocation by post. You will find our address on the cover letter.

Who evaluates the data?

The questionnaire is evaluated exclusively by staff of the Department of Health Care Management at the Technische Universität Berlin (Head: Prof. Dr. med. Reinhard Busse, MPH).

Do you have to participate in the survey?

Your participation is voluntary. If you do not wish to answer a question, we understand. If you find that you do not want to or cannot answer a large part of the questions, we ask you to refrain from participating.

How do I participate in the raffle for twenty 50 € shopping vouchers?

If you would like to take part in the raffle for the shopping vouchers, please make the appropriate entries in the raffle form. Please place the raffle form in the separate small envelope for the raffle form, seal it and place it in the enclosed return envelope. The winners will be drawn at random. Only those participants who return a questionnaire to us will be entered into the draw.

Who can you contact if you have any questions?

If you have any questions, e.g., about the questionnaire or the research project, please do not hesitate to contact the project staff Susanne Felgner (susanne.felgner@tu-berlin.de) or Dr. rer. oec. Cornelia Henschke (cornelia.henschke.1@tu-berlin.de) by e-mail or by phone **030/314 28701**. If you have any questions about your address data stored in the population register, please contact your residents' registration office.

The questionnaire is divided into 3 parts A, B and C below.

Part A: Explanation of treatment and importance of attributes

Part A1: Explanation of treatment

A full crown (i.e., a full dental crown) is a dental prosthesis in which an artificial crown is placed over a tooth like cap. Different materials are used, such as metal or ceramic, which consequently have different attributes (color, durability, etc.). A full crown is used, for example, in the case of tooth breakage after an accident, for follow-up treatment after root inflammation or for aesthetic reasons.

In the following, we will only use the term **dental crown**.

If a dental crown is not inserted, although it would be medically necessary, it is possible that the tooth **will become discolored,** or the patient **will lose the tooth** altogether.

If the dental crown is medically necessary, your health insurance will pay half of the total costs of a defined treatment (so-called standard treatment). If a bonus booklet is kept or a hardship case application is made, the co-payment by the health insurance company may be increased. If patients have a supplementary dental insurance, the amount they must pay themselves depends on the benefits provided by the insurance.

Dental areas

The design of the standard treatment and the amount of costs covered by the statutory health insurance depend on the position of the tooth in the mouth. A differentiation is made between **"non-visible tooth area"** and **"visible tooth area"** (dental scheme, see below).

Dental scheme:

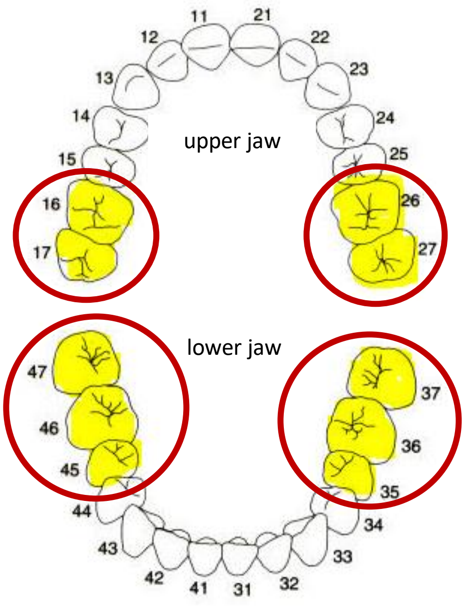

"non-visible tooth area";
teeth 16, 17, 26, 27, 45–47 and 35–37
(colored and circled in the picture)

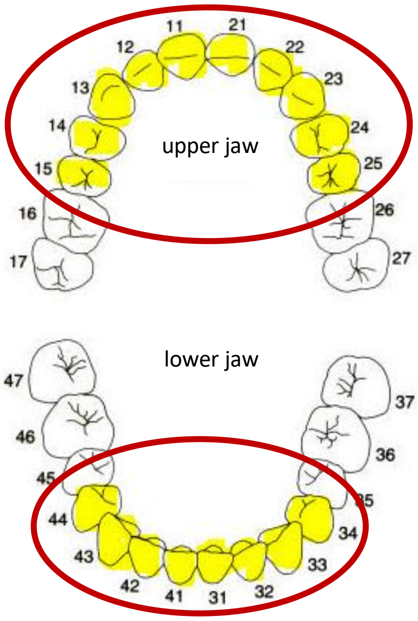

"visible tooth area",
teeth 15–25 and 34–44
(colored and circled in the picture)

Part A2: Treatment attributes and levels

When your dentist suggests dental crown treatment, the treatment attributes may be of varying importance to you. In this questionnaire, we look at certain treatment attributes of dental crowns. The treatment attributes are: **1. aesthetics**, **2. compatibility**, **3. durability**, and **4. out-of-pocket payment** and those can take different levels, i.e., the durability of a dental crown can be 5 or 10 years, etc.

In the following table, we define the treatment attributes and indicate which levels those can take:

**Attribute**	Definition of attribute given to participants, and levels of attribute
**1. Aesthetics**	In terms of appearance, result of treatment individually perceived as beautiful. This attribute describes the visibility of a dental crown.
	**levels:** not visible, lightly visible, strongly visible
**2. Compatibility**	Intolerance reaction of human body due to dental material in form of an allergic or a local toxic reaction[1].
	**levels:** no risk, 1 out of 10,000 people with allergic or local toxic reaction
**3. Durability**	Expected length of time from completion of a treatment to another new treatment that is medically or technically necessary.
	**levels:** 5 years, 10 years, 15 years, 25 years
**4. Out-of-pocket payment**	Costs that must be paid by patient for dental crown treatment. The co-payment taken by health insurance has already been subtracted here.
	**levels in "non-visible-tooth area":** 50 €, 150 €, 450 €, 600 €<br>**levels in "visible-tooth area":** 50 €, 200 €, 450 €, 600 €

PLEASE NOTE that, looking at the treatment attributes, we are NOT describing a CERTAIN TREATMENT or a CERTAIN MATERIAL used for dental crowns! We just want to know how important the attribute is to you.

For a better understanding we visualized the attribute aesthetics of a dental crown in the attached extra document **Presentation of the treatment attribute "Aesthetics"**.

[1] In very rare cases (1 in 10,000 people), an intolerance reaction, i.e., an allergic or local toxic reaction, may occur. **Allergies** are characterized by symptoms such as dry mouth, toothache, and receding gums to discomfort in the throat, lip eczema or rash on the face. **Local toxic reactions** are non-allergic inflammations of the oral mucosa in the immediate vicinity of the tooth crown. In the cases described, depending on the severity of the clinical symptoms, the "problematic material" must be replaced or (the dental crown) removed completely.

Technische Universität Berlin

Part B: Importance of treatment attributes

In the following, we would like to know how important certain treatment attributes of a dental crown are to you in the "non-visible tooth area" and in the "visible tooth area". Imagine that your dentist suggests a dental crown treatment.

First, on a scale from "very important" to "not important", we ask you to determine the importance of the attributes **1. aesthetics**, **2. compatibility**, **3. durability** and **4. out-of-pocket payment** from your perspective by placing a cross in the appropriate box.

We then present you a total of 18 different choice situations in which **"Treatment A"** and **"Treatment B"** are given with its attributes and in various levels. Please mark which treatment you would choose at the dentist by placing a cross in the appropriate box. If neither of the two treatment alternatives appeals to you and you would not opt for any treatment at the dentist, please place your cross in the **"none of the treatments"** box.

Only these alternatives are given. If you do not choose any of the treatment alternatives, it means that you will not get suggestions and a treatment from another dentist.

See an **example** for the "non-visible teeth area" here:

**Attributes**	**Treatment A**	**Treatment B**
1. Aesthetics	lightly visible	strongly visible
2. Compatibility	no risk	1 out of 10,000 people with allergic or local toxic reaction
3. Durability	10 years	5 years
4. Out-of-pocket payment	450 €	150 €
**I choose ...**	... treatment A. ☐	... treatment B. ☒
	... none of the treatments. ☐	

Decision in words:

You have placed a cross at the box "Treatment B" and have thus opted ***for "Treatment B"***. This means that if the dentist suggests a dental crown for the "non-visible tooth area", you opt for the crown that is strongly visible, where an allergic or local toxic reaction occurs in 1 in 10,000 people, which is expected to last 5 years and for which you must pay 150 € out of your own pocket. The co-payment from your health insurance company has already been deducted from this amount. If you have a supplementary dental insurance, your out-of-pocket payment may be reduced. This depends on the benefits of your supplementary dental insurance.

You have decided ***against "Treatment A"*** and said that the choice ***"none of the treatments" does not apply to you***.

Technische Universität Berlin

Part B1: "Non-visible tooth area"

All questions and decisions in Part B1 relate exclusively to the "non-visible tooth area".

Please tell us in the following how important the attributes 1. aesthetics, 2. compatibility, 3. durability and 4. out-of-pocket payment are to you, by placing a cross in the appropriate box!

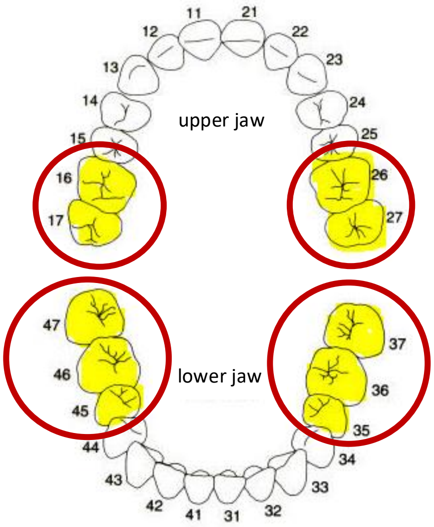

1. Aesthetics

The attribute **aesthetics** for a dental crown in the "non-visible tooth area" is… to me.				
… very important	… important	… moderately important	… less important	… not important
☐	☐	☐	☐	☐

2. Compatibility

The attribute **compatibility** for a dental crown in the "non-visible tooth area" is… to me.				
… very important	… important	… moderately important	… less important	… not important
☐	☐	☐	☐	☐

3. Durability

The attribute **durability** for a dental crown in the "non-visible tooth area" is… to me.				
… very important	… important	… moderately important	… less important	… not important
☐	☐	☐	☐	☐

4. Out-of-pocket payment

The attribute **out-of-pocket payment** for a dental crown in the "non-visible tooth area" is… to me.				
… very important	… important	… moderately important	… less important	… not important
☐	☐	☐	☐	☐

In the following, different treatment alternatives, that the dentist proposes to you, are presented to you in pairs one after the other. **Please place a cross in the box of the alternative you choose, i.e., "Treatment A" or "Treatment B".** If neither of the two treatments appeals to you, please place your cross in the box **"none of the treatments"**.

Only these alternatives are given. If you do not choose any of the treatment alternatives, it means that you do not want to get suggestions and a treatment from another dentist.

The alternatives refer exclusively to teeth of the "non-visible tooth area".

1. Choice "Non-visible tooth area"

**Attributes**	**Treatment A**	**Treatment B**
1. Aesthetics	strongly visible	not visible
2. Compatibility	1 out of 10,000 people with allergic or local toxic reaction	no risk
3. Durability	10 years	25 years
4. Out-of-pocket payment	50 €	150 €
**I choose …**	… treatment A. ☐	… treatment B. ☐
	… none of the treatments. ☐	

2. Choice "Non-visible tooth area"

**Attributes**	**Treatment A**	**Treatment B**
1. Aesthetics	not visible	lightly visible
2. Compatibility	no risk	1 out of 10,000 people with allergic or local toxic reaction
3. Durability	10 years	25 years
4. Out-of-pocket payment	450 €	600 €
**I choose …**	… treatment A. ☐	… treatment B. ☐
	… none of the treatments. ☐	

3. Choice "Non-visible tooth area"		
**Attributes**	**Treatment A**	**Treatment B**
1. Aesthetics	lightly visible	not visible
2. Compatibility	no risk	1 out of 10,000 people with allergic or local toxic reaction
3. Durability	25 years	10 years
4. Out-of-pocket payment	600 €	150 €
**I choose …**	… treatment A. ☐	… treatment B. ☐
	… none of the treatments. ☐	

4. Choice "Non-visible tooth area"		
**Attributes**	**Treatment A**	**Treatment B**
1. Aesthetics	lightly visible	strongly visible
2. Compatibility	1 out of 10,000 people with allergic or local toxic reaction	no risk
3. Durability	5 years	10 years
4. Out-of-pocket payment	450 €	50 €
**I choose …**	… treatment A. ☐	… treatment B. ☐
	… none of the treatments. ☐	

5. Choice "Non-visible tooth area"		
**Attributes**	**Treatment A**	**Treatment B**
1. Aesthetics	not visible	strongly visible
2. Compatibility	no risk	1 out of 10,000 people with allergic or local toxic reaction
3. Durability	5 years	15 years
4. Out-of-pocket payment	150 €	600 €
**I choose …**	… treatment A. ☐	… treatment B. ☐
	… none of the treatments. ☐	

6. Choice "Non-visible tooth area"		
**Attributes**	**Treatment A**	**Treatment B**
1. Aesthetics	strongly visible	lightly visible
2. Compatibility	1 out of 10,000 people with allergic or local toxic reaction	no risk
3. Durability	15 years	5 years
4. Out-of-pocket payment	600 €	50 €
**I choose …**	… treatment A. ☐	… treatment B. ☐
	… none of the treatments. ☐	

7. Choice "Non-visible tooth area"		
**Attributes**	**Treatment A**	**Treatment B**
1. Aesthetics	lightly visible	strongly visible
2. Compatibility	1 out of 10,000 people with allergic or local toxic reaction	no risk
3. Durability	5 years	15 years
4. Out-of-pocket payment	50 €	450 €
**I choose …**	… treatment A. ☐	… treatment B. ☐
	… none of the treatments. ☐	

8. Choice "Non-visible tooth area"		
**Attributes**	**Treatment A**	**Treatment B**
1. Aesthetics	strongly visible	not visible
2. Compatibility	no risk	1 out of 10,000 people with allergic or local toxic reaction
3. Durability	25 years	15 years
4. Out-of-pocket payment	150 €	450 €
**I choose …**	… treatment A. ☐	… treatment B. ☐
	… none of the treatments. ☐	

Part B2: "Visible tooth area"

All questions and decisions in Part B2 relate exclusively to the to the "visible tooth area".

Please tell us in the following how important the attributes 1. aesthetics, 2. compatibility, 3. durability and 4. out-of-pocket payment are to you, by placing a cross in the appropriate box!

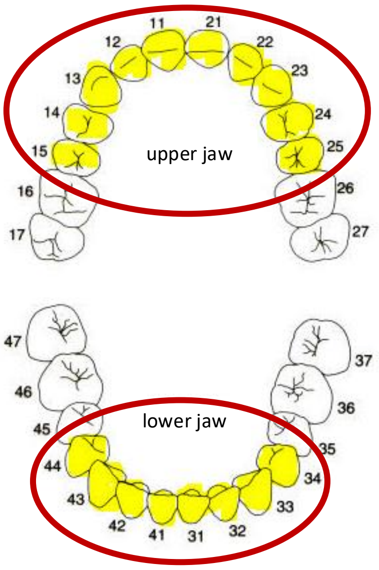

1. Aesthetics

The attribute **aesthetics** for a dental crown in the "visible tooth area" is… to me.				
… very important	… important	… moderately important	… less important	… not important
☐	☐	☐	☐	☐

2. Compatibility

The attribute **compatibility** of a dental crown in the "visible tooth area" is… to me.				
… very important	… important	… moderately important	… less important	… not important
☐	☐	☐	☐	☐

3. Durability

The attribute **durability** of a dental crown in the "visible tooth area" is… to me.				
… very important	… important	… moderately important	… less important	… not important
☐	☐	☐	☐	☐

4. Out-of-pocket payment

The attribute **out-of-pocket payment** of a dental crown in the "visible tooth area" is… to me.				
… very important	… important	… moderately important	… less important	… not important
☐	☐	☐	☐	☐

In the following, different treatment alternatives, that the dentist proposes to you, are presented to you in pairs one after the other. **Please place a cross in the box of the alternative you choose, i.e., "Treatment A" or "Treatment B".** If neither of the two treatments appeals to you, please place your cross in **"none of the treatments"**.

Only these alternatives are given. If you do not choose any of the treatment alternatives, it means that you do not want to get suggestions and a treatment from another dentist.

The alternatives refer exclusively to teeth from the "visible tooth area".

1. Choice "Visible tooth area"

**Attributes**	**Treatment A**	**Treatment B**
1. Aesthetics	strongly visible	not visible
2. Compatibility	1 out of 10,000 people with allergic or local toxic reaction	no risk
3. Durability	10 years	25 years
4. Out-of-pocket payment	50 €	200 €
**I choose …**	… treatment A. ☐	… treatment B. ☐
	… none of the treatments. ☐	

2. Choice "Visible tooth area"

**Attributes**	**Treatment A**	**Treatment B**
1. Aesthetics	not visible	lightly visible
2. Compatibility	no risk	1 out of 10,000 people with allergic or local toxic reaction
3. Durability	10 years	25 years
4. Out-of-pocket payment	450 €	600 €
**I choose …**	… treatment A. ☐	… treatment B. ☐
	… none of the treatments. ☐	

3. Choice "Visible tooth area"		
**Attributes**	**Treatment A**	**Treatment B**
1. Aesthetics	lightly visible	not visible
2. Compatibility	no risk	1 out of 10,000 people with allergic or local toxic reaction
3. Durability	25 years	10 years
4. Out-of-pocket payment	600 €	200 €
**I choose …**	… treatment A. ☐	… treatment B. ☐
	… none of the treatments. ☐	

4. Choice "Visible tooth area"		
**Attributes**	**Treatment A**	**Treatment B**
1. Aesthetics	lightly visible	strongly visible
2. Compatibility	1 out of 10,000 people with allergic or local toxic reaction	no risk
3. Durability	5 years	10 years
4. Out-of-pocket payment	450 €	50 €
**I choose …**	… treatment A. ☐	… treatment B. ☐
	… none of the treatments. ☐	

5. Choice "Visible tooth area"		
**Attributes**	**Treatment A**	**Treatment B**
1. Aesthetics	not visible	strongly visible
2. Compatibility	no risk	1 out of 10,000 people with allergic or local toxic reaction
3. Durability	5 years	15 years
4. Out-of-pocket payment	200 €	600 €
**I choose …**	… treatment A. ☐	… treatment B. ☐
	… none of the treatments. ☐	

6. Choice "Visible tooth area"		
**Attributes**	**Treatment A**	**Treatment B**
1. Aesthetics	strongly visible	lightly visible
2. Compatibility	1 out of 10,000 people with allergic or local toxic reaction	no risk
3. Durability	15 years	5 years
4. Out-of-pocket payment	600 €	50 €
**I choose …**	… treatment A. ☐	… treatment B. ☐
	… none of the treatments. ☐	

7. Choice "Visible tooth area"		
**Attributes**	**Treatment A**	**Treatment B**
1. Aesthetics	lightly visible	strongly visible
2. Compatibility	1 out of 10,000 people with allergic or local toxic reaction	no risk
3. Durability	5 years	15 years
4. Out-of-pocket payment	50 €	450 €
**I choose …**	… treatment A. ☐	… treatment B. ☐
	… none of the treatments. ☐	

8. Choice "Visible tooth area"		
**Attributes**	**Treatment A**	**Treatment B**
1. Aesthetics	strongly visible	not visible
2. Compatibility	no risk	1 out of 10,000 people with allergic or local toxic reaction
3. Durability	25 years	15 years
4. Out-of-pocket payment	200 €	450 €
**I choose …**	… treatment A. ☐	… treatment B. ☐
	… none of the treatments. ☐	

9. Choice "Visible tooth area"		
**Attributes**	**Treatment A**	**Treatment B**
1. Aesthetics	strongly visible	not visible
2. Compatibility	1 von 10,000 Personen mit Allergie oder lokaltoxischer Reaktion	no risk
3. Durability	5 years	25 years
4. Out-of-pocket payment	600 €	50 €
**I choose …**	… treatment A. ☐	… treatment B. ☐
	… none of the treatments. ☐	

10. Choice "Visible tooth area"		
**Attributes**	**Treatment A**	**Treatment B**
1. Aesthetics	lightly visible	not visible
2. Compatibility	no risk	1 out of 10,000 people with allergic or local toxic reaction
3. Durability	25 years	10 years
4. Out-of-pocket payment	600 €	200 €
**I choose …**	… treatment A. ☐	… treatment B. ☐
	… none of the treatments. ☐	

Part C: Participants‘ characteristics

Questions about yourself

[1] How old are you (in age groups)?

- □ under 30 years
- □ 30 to 34 years
- □ 35 to 39 years
- □ 40 to 44 years
- □ 45 to 49 years
- □ 50 to 54 years
- □ 55 to 59 years
- □ 60 to 64 years
- □ 65 to 69 years
- □ 70 to 74 years
- □ 75 to 79 years
- □ 80 to 84 years
- □ 85 to 89 years
- □ 90 years and older

[2] What is your gender?

- □ female
- □ male
- □ other

[3] What is your highest level of education?

- □ (technical) university degree
- □ vocational training
- □ (technical) A-level
- □ high school diploma (10 years)
- □ high school diploma (9 years)
- □ no school-leaving qualification
- □ other ___________________________________

[4] Are you currently employed?

- □ yes, I am full-time employed
- □ yes, I am part-time employed
- □ I am a college student (not employed)
- □ no, I am unemployed
- □ no, I am retired due to illness
- □ no, I am retired due to age
- □ other ___________________________________

[5] What is your monthly household (net) income, i.e., how much money is available to your entire household (including income from your partner, for example) each month?

- □ under 500 €
- □ 500 to under 750 €
- □ 750 to under 1,000 €
- □ 1,000 to under 1,500 €
- □ 1,500 to under 2,500 €
- □ 2,500 to under 3,500 €
- □ 3,500 to under 4,500 €
- □ 4,500 to under 5,500 €
- □ 5,500 to under 6,500 €
- □ over 6,500 €

[6] In which type of city or municipality do you live?

- □ large city (> 100,000 inhabitants)
- □ medium-sized city (> 20,000 – 100,000 inhabitants)
- □ small town (> 5,000 – 20,000 inhabitants)
- □ rural community / village (< 5,000 inhabitants)
- □ I do not know

[7] In which administrative district of Berlin or which city do you live?

- □ Berlin Charlottenburg-Wilmersdorf
- □ Berlin Friedrichshain-Kreuzberg
- □ Berlin Lichtenberg
- □ Berlin Marzahn-Hellersdorf
- □ Berlin Mitte
- □ Berlin Neukölln
- □ Berlin Pankow
- □ Berlin Reinickendorf
- □ Berlin Spandau
- □ Berlin Steglitz-Zehlendorf
- □ Berlin Tempelhof-Schöneberg
- □ Berlin Treptow-Köpenick
- □ Brandenburg an der Havel, City
- □ Cottbus, City
- □ Frankfurt (Oder), City
- □ Potsdam, City
- □ other _______________________________
- □ I do not know

Questions about your insurance coverage

[8] Do you have statutory or private health insurance (SHI – statutory health insurance, PHI – private health insurance)?

□ SHI
□ PHI
□ I do not know

[9] Do you have supplementary dental insurance?

□ yes
□ no
□ I do not know

[10] Do you have a **bonus booklet**[2]?

□ yes
□ no
□ I do not know

a. If yes: Have you ever submitted a bonus booklet with at least 5 stamps in a row to your health insurance company, so that the coverage is extended by the health insurance company?

□ yes
□ no
□ I do not know

[11] Would you be considered as a **hardship case**[3]?

□ yes
□ no
□ I do not know

a. If yes: Have you ever made use of the hardship case provision for yourself?

□ yes
□ no
□ I do not know

---

[2] The **bonus booklet** is a small paper booklet in which your annual preventive check-up at the dentist is confirmed with a stamp. If you received proof 5 stamps in a row, coverage of health insurances for dentures (e.g., dental crown) increases to 60% of the total costs of the standard treatment. With 10 stamps in a row, coverage of the health insurance even increases to 65%. Change in the law: As of 10/01/2020, the coverage of the health insurance company is at least 60 % (or 70, or 75 %; for 5, or 10 stamps).

[3] Patients can submit a **hardship case** application to their health insurance company if their gross monthly (family) income is below or just above a certain limit (For 2020, the following applies: single person 1,274 €; with one relative 1,751.75 € and each additional relative an additional 318.50 €). Other groups of people who can be classified as hardship cases include, for example, students who receive federal education advancement grants *(short, German: BAföG)*. In a case of hardship, the health insurance company will pay up to 100% of the costs of the standard treatment. This means that the patient's own payment, which must be paid out of pocket, may not apply at all.

Technische Universität Berlin

Questions about your oral health

[12] How would you rate your overall oral health? Please rate on a scale of 1 (very good) to 5 (very poor).

1 (very good)	2 (good)	3 (moderate)	4 (poor)	5 (very poor)
☐	☐	☐	☐	☐

If you do not have an answer, simply <u>do not</u> place a cross.

[13] Have you ever received a dental crown, e.g., after root canal treatment?

☐ yes
☐ no
☐ I do not know

[14] Have you ever decided against a dental crown treatment at the dentist?

☐ yes
☐ no
☐ I do not know

a. If yes: What was the reason for deciding against it?

☐ costs too high
☐ fear of treatment
☐ no trust in dentist
☐ I think, it was not necessary
☐ other ___________________________________

[15] **<u>Change in the law:</u>** As of 10/01/2020, the health insurance company will pay at least 60% of the total costs of standard treatment instead of the previous 50% (cf. full crown in the non-visible tooth area: **new 194.34 €**, previously 161.95 € paid by health insurance company). Does this change alter your decision for or against dental crown treatment?

☐ yes
☐ no
☐ I do not know

a. If yes: To what extent does your decision change?

☐ I then decide **for a <u>standard care treatment</u>**
☐ I then decide **for a treatment <u>beyond standard care</u>**, e.g., full ceramic crown

[16] Does your health status (e.g., constitution, allergy) permit dental crown treatment?

☐ yes
☐ no
☐ I do not know

[17] If dental crowns are strongly visible: Does your decision **<u>against</u>** a treatment alternative depends on the color (e.g., gold-colored) of the dental crown?

☐ yes
☐ no, I **<u>always decide against</u>** a strongly visible dental crown
☐ I do not know

a. If yes: Which color of dental crown would you **choose** as treatment alternative? (Multiple answers possible)

◻ color golden-metal
◻ color light grey metallic
◻ color dark grey metallic
◻ I do not know

Please rate the questionnaire

[18] How difficult was it for you to decide between the treatment alternatives? Please rate using a scale from 1 (very easy) to 5 (very difficult).

1 (very easy)	2 (easy)	3 (moderate)	4 (difficult)	5 (very difficult)
◻	◻	◻	◻	◻

If you do not have an answer, simply do not place a cross.

[19] How easy or how difficult did you find it to complete the questionnaire? Please rate using a scale from 1 (very easy) to 5 (very difficult).

1 (very easy)	2 (easy)	3 (moderate)	4 (difficult)	5 (very difficult)
◻	◻	◻	◻	◻

If you do not have an answer, simply do not place a cross.

[20] How much time did it take to complete the questionnaire? ___________ minutes

[21] Do you have any other comments, advice, etc.?

____

____

____

____

____

____

____

____

____

____

____

We thank you very much for your cooperation!

Please separate the first sheet with your address from the questionnaire. Please fold the filled-out questionnaire in half and return it to us in the enclosed envelope by dropping it in a Deutsche Post mailbox. The postage will be paid by the Technische Universität Berlin.

Technische Universität Berlin
