# Supplementary material for: Patients’ preferences in dental care: A discrete-choice experiment and an analysis of willingness-to-pay
Source: PLoS One. 2023 Feb 27;18(2):e0280441. doi: 10.1371/journal.pone.0280441 (PMC9970100; doi:10.1371/journal.pone.0280441)
Supplement: S2 File — (PDF) [file pone.0280441.s002.pdf]

Technische Universität Berlin

Presentation of the treatment attribute "Aesthetics"

Here we visualized the attribute aesthetics of a dental crown (red arrow) and its levels.
<u>You may place this document next to the questionnaire when filling in. This may make it easier for you to imagine an alternative treatment.</u>

"Non-visible tooth area" (teeth 16, 17, 26, 27, 45–47 and 35–37 in dental scheme)

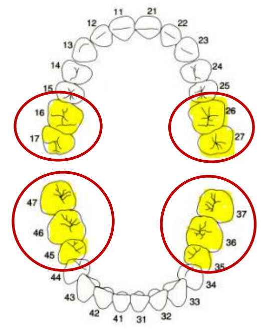

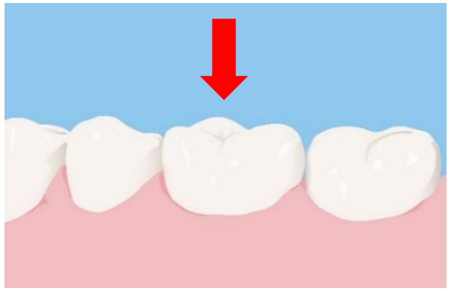

Natural color

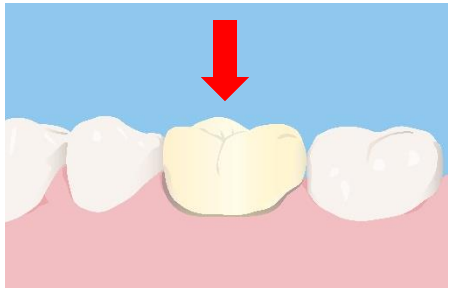

Lightly visible

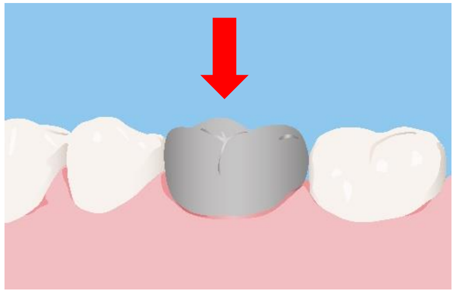

Strongly visible

"Visible tooth area" (teeth 15–25 and 34–44 in dental scheme)

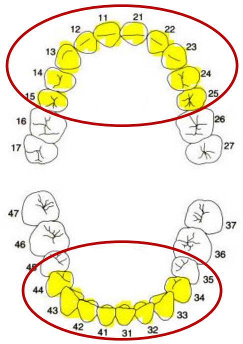

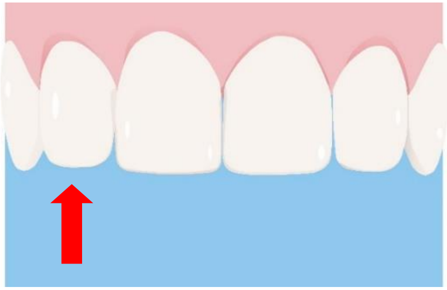

Natural color

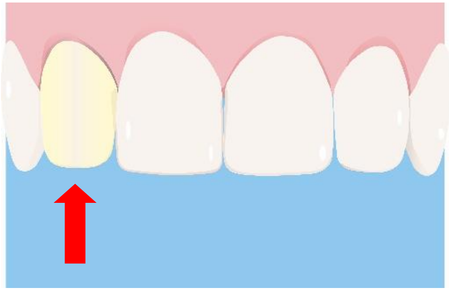

Lightly visible

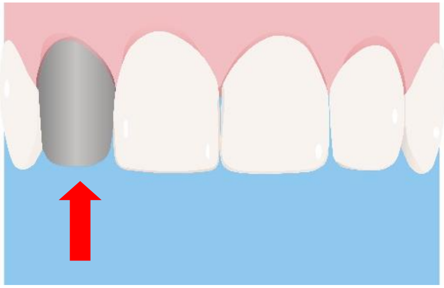

Strongly visible

PLEASE NOTE. Using these treatment levels, we are NOT describing ANY CERTAIN TREATMENT or ANY CERTAIN MATERIAL that is common for dental crowns!
We only want to know how important treatment levels are to you.
