# Supplementary figures and images for: Patients’ preferences in dental care: A discrete-choice experiment and an analysis of willingness-to-pay
Source: PLoS One. 2023 Feb 27;18(2):e0280441. doi: 10.1371/journal.pone.0280441 (PMC9970100; doi:10.1371/journal.pone.0280441)

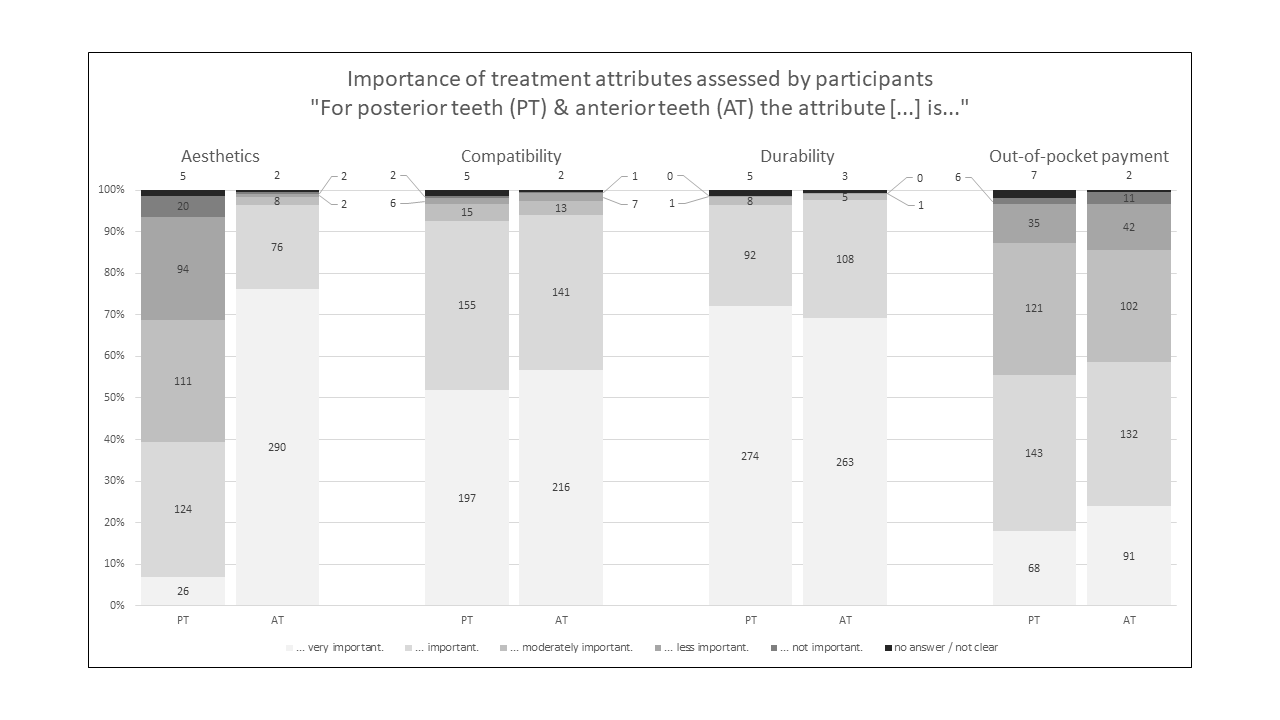

Supplement: S6 File — (TIF) [file pone.0280441.s006.tif]

**S1 Table. Design output.**

SAS, with RESTRICTIONS


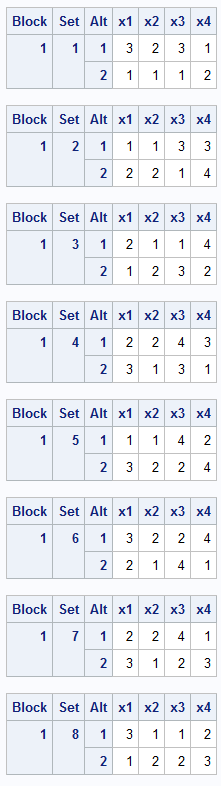


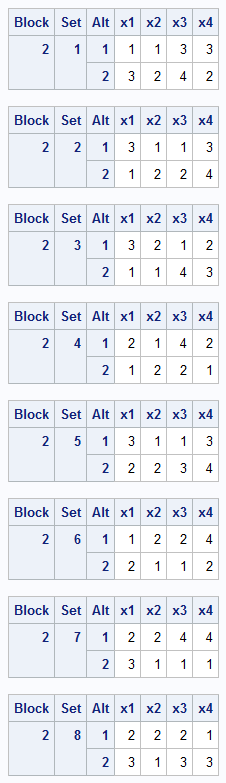


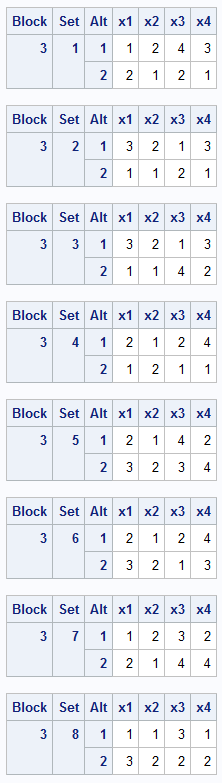


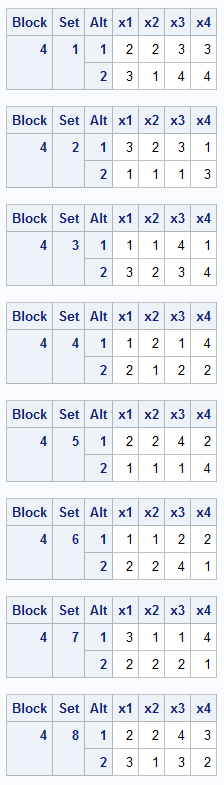


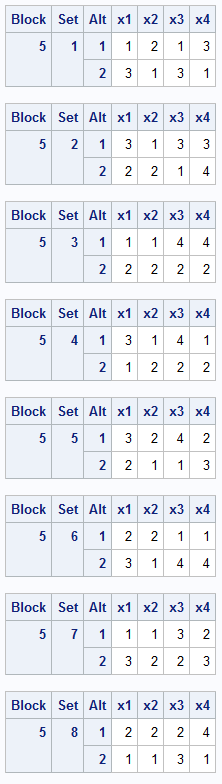


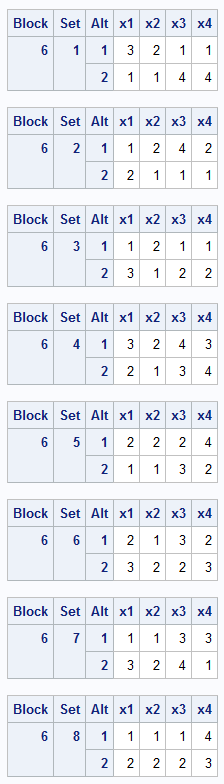


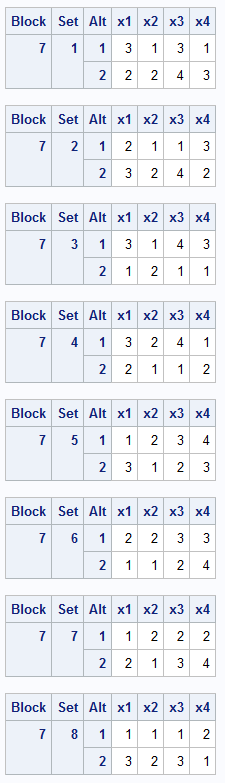


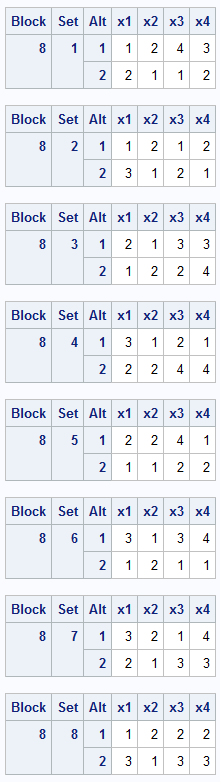


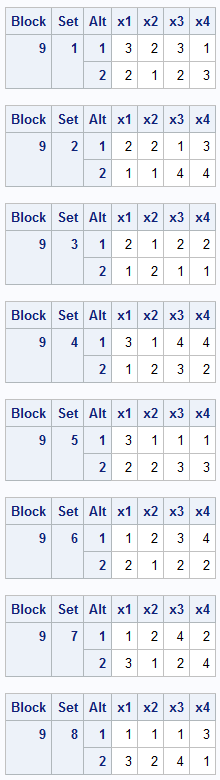


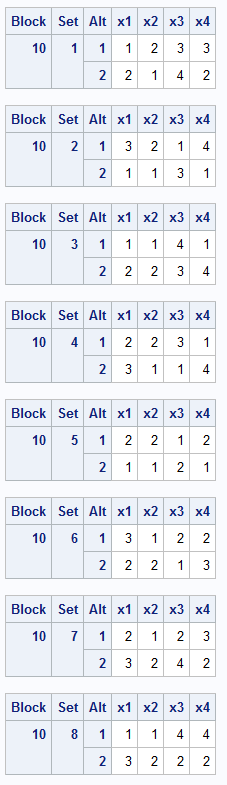


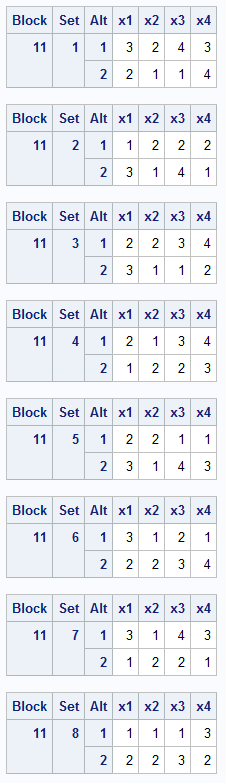


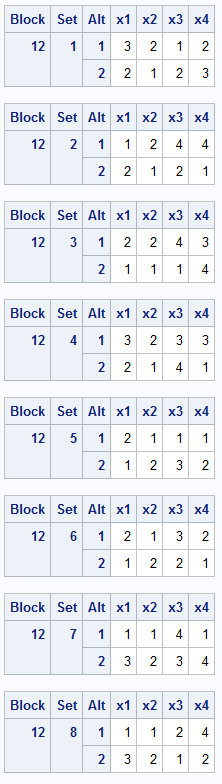

Supplement: S1 Table — (DOCX) [file pone.0280441.s008.docx]
