# Supplementary material for: Patients’ preferences in dental care: A discrete-choice experiment and an analysis of willingness-to-pay
Source: PLoS One. 2023 Feb 27;18(2):e0280441. doi: 10.1371/journal.pone.0280441 (PMC9970100; doi:10.1371/journal.pone.0280441)
Supplement: S7 File — (DOCX) [file pone.0280441.s007.docx]

**S7 File. Regression plots on WTPmax and SHI+ analysis.**

**(I) Regression plot on WTPmax and age (PT)**


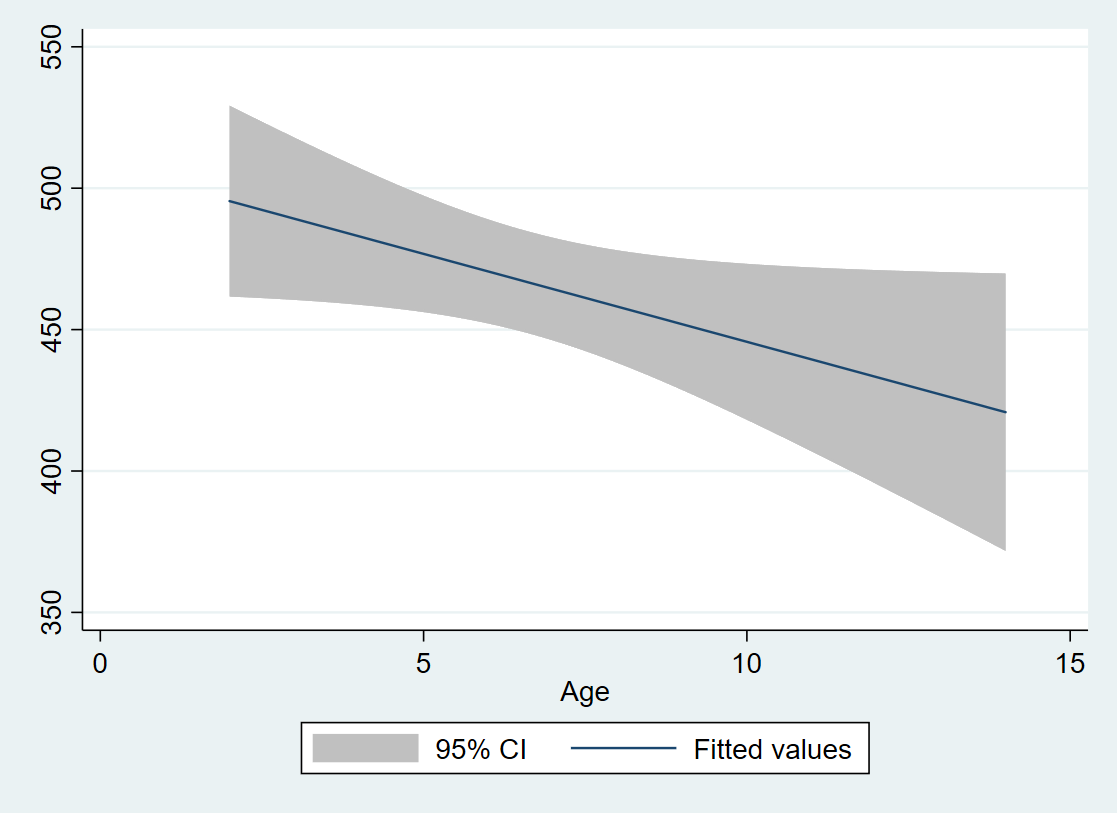


**(II) Regression plot on WTPmax and age (AT)**


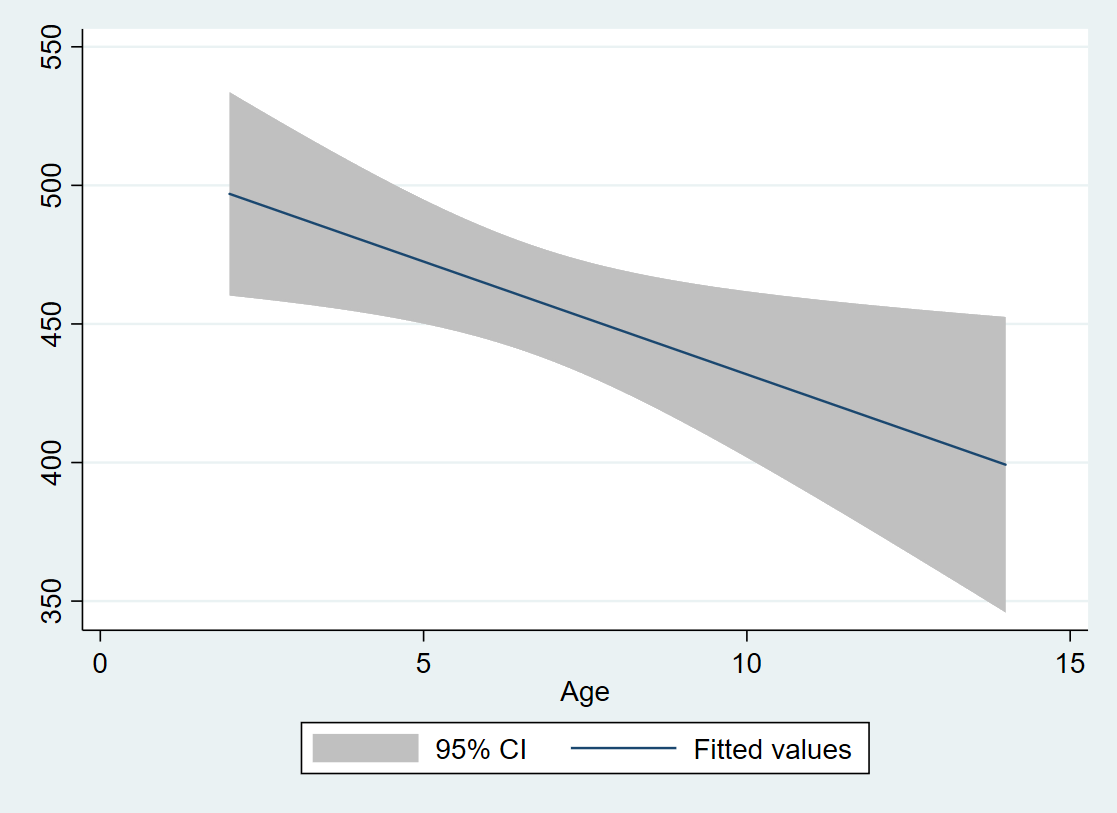


**(III) Regression plot on SHI+ variable and importance of aesthetics (PT)**


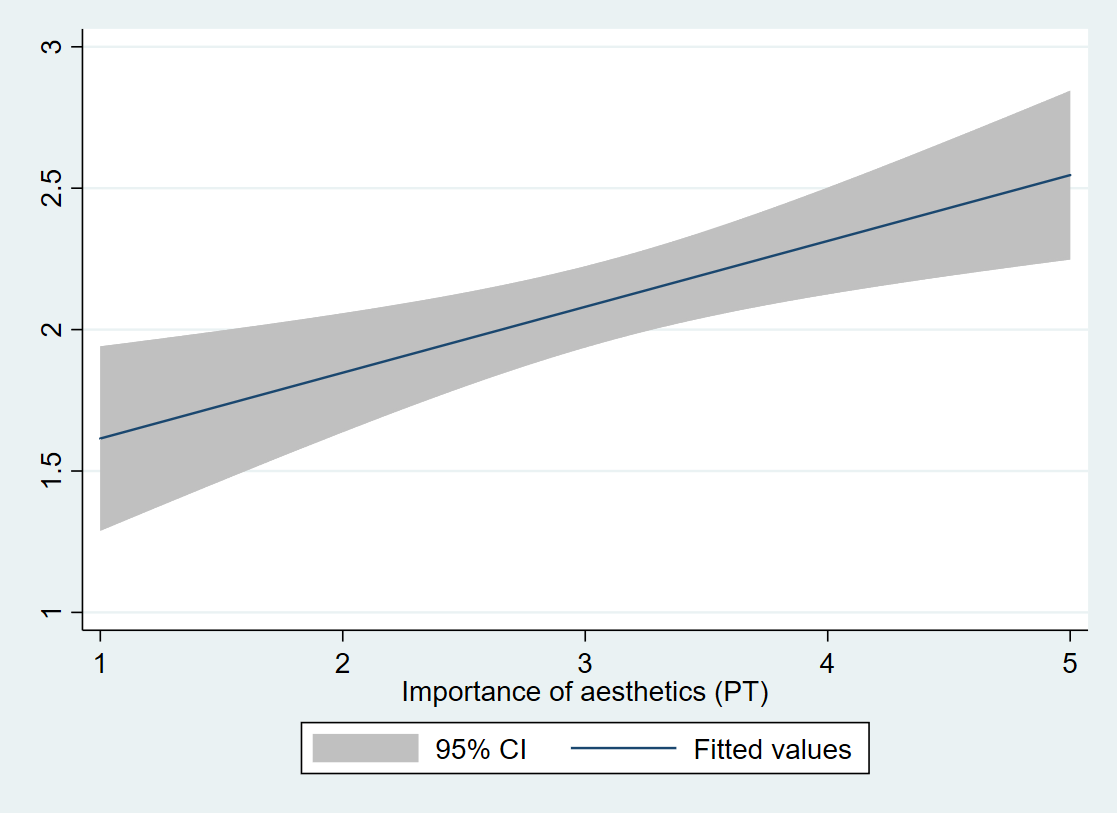


**(IV) Regression plot on SHI+ variable and importance of out-of-pocket payments (PT)**


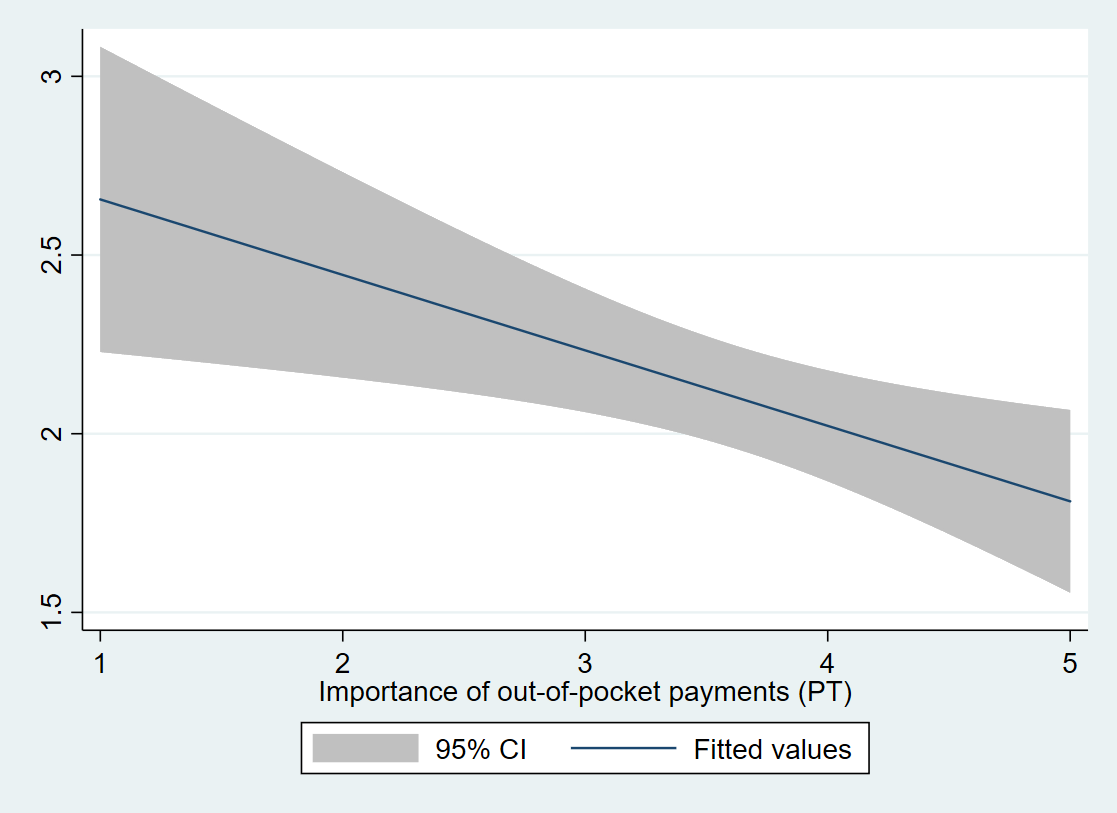


**(V) Regression plot on SHI+ variable and age (AT)**


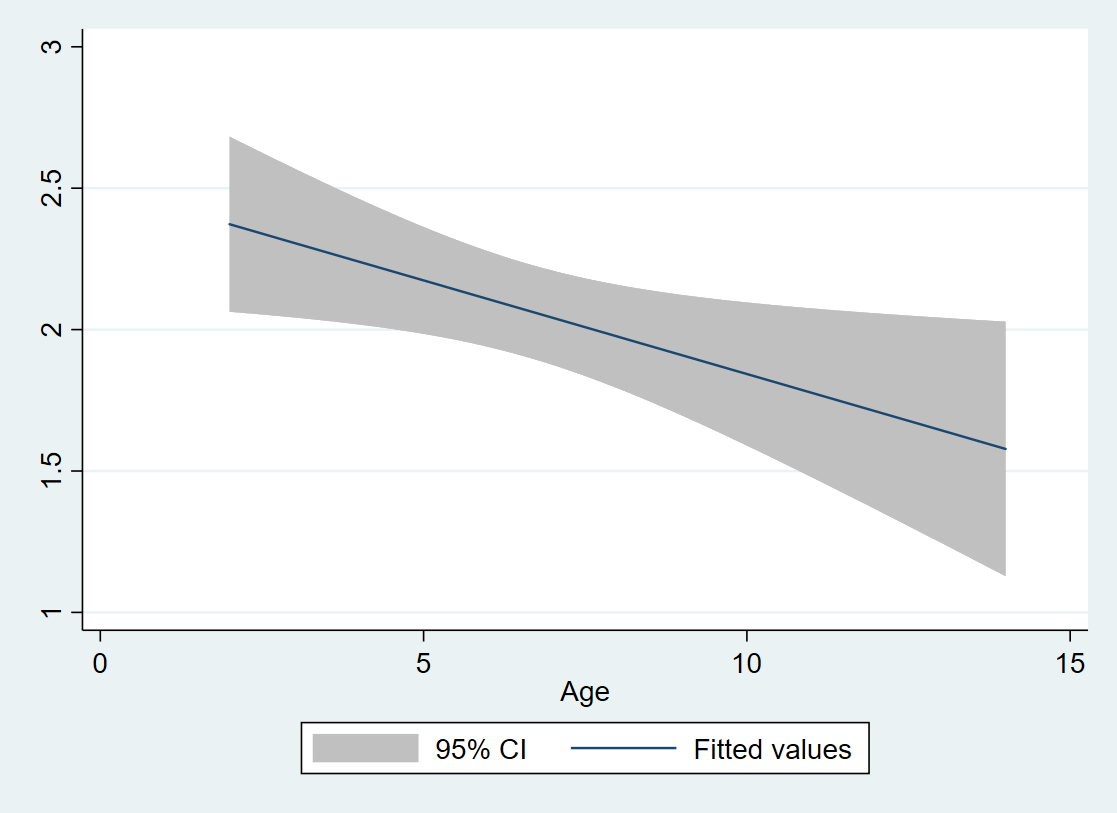


**(VI) Regression plot on SHI+ variable and urban & rural region (AT)**


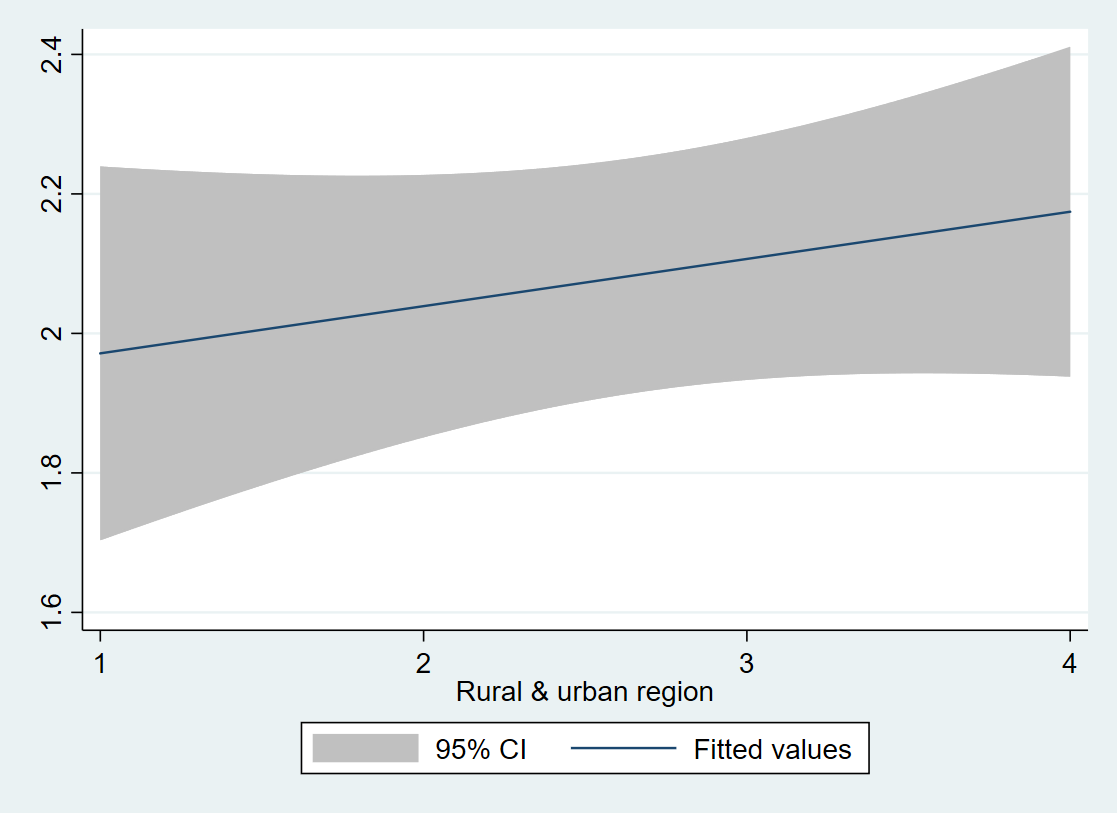


**(VII) Regression plot on SHI+ variable and importance of out-of-pocket payments (AT)**


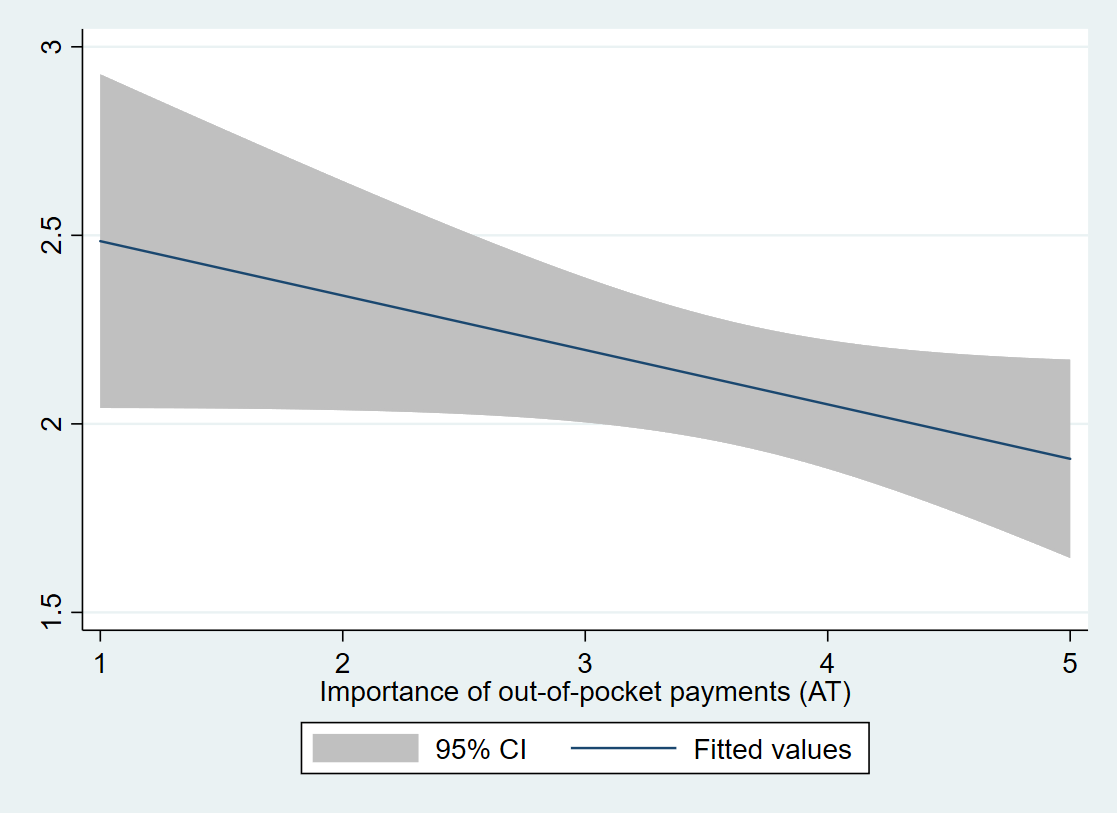


Abbreviation:

WTPmax – highest level value of the attribute "out-of-pocket payment" for a chosen treatment alternative across all alternatives per participant presenting maximum individual willingness-to-pay (WTP)

SHI+ – treatment presenting attribute levels beyond statutory health insurance (SHI) standard care

PT – posterior teeth

AT – anterior teeth

CI – confidence interval
