# Supplementary material for: Patients’ preferences in dental care: A discrete-choice experiment and an analysis of willingness-to-pay
Source: PLoS One. 2023 Feb 27;18(2):e0280441. doi: 10.1371/journal.pone.0280441 (PMC9970100; doi:10.1371/journal.pone.0280441)
Supplement: S2 Table — (DOCX) [file pone.0280441.s009.docx]

**S2 Table. Calculation of D-efficiency.**

| SAS System |
| --- |

| Design Summary    Number of  Levels Frequency    2 1  3 1  4 2 |
| --- |

| SAS System |
| --- |

| Saturated = 25  Full Factorial = 96    Some Reasonable Cannot Be  Design Sizes Violations Divided By    96 * 0  48 1 32  72 3 16 32 48  32 6 3 6 12 24 48  36 6 8 16 24 32 48  60 6 8 16 24 32 48  64 6 3 6 12 24 48  84 6 8 16 24 32 48  80 7 3 6 12 24 32 48  40 8 3 6 12 16 24 32 48  25 S 13 2 3 4 6 8 12 16 24 32 48    * - 100% Efficient design can be made with the MktEx macro.  S - Saturated Design - The smallest design that can be made.  Note that the saturated design is not one of the  recommended designs for this problem. It is shown  to provide some context for the recommended sizes. |
| --- |

| SAS System |
| --- |

| Algorithm Search History |
| --- |

| Current Best  Design Row,Col D-Efficiency D-Efficiency Notes  ----------------------------------------------------------  1 Start 100.0000 100.0000 Tab  1 End 100.0000 |
| --- |

| SAS System |
| --- |

| The OPTEX Procedure    Class Level Information    Class Levels Values    x1 3 1 2 3  x2 2 1 2  x3 4 1 2 3 4  x4 4 1 2 3 4 |
| --- |

| SAS System |
| --- |

| **Design-Number** | **D-** **efficiency** | **A-** **efficiency** | **G-** **efficiency** | **Avg. forecast standard error** |
| --- | --- | --- | --- | --- |
| **1** | 100.0000 | 100.0000 | 100.0000 | 0.5103 |
