# Supplementary material for: Patients’ preferences in dental care: A discrete-choice experiment and an analysis of willingness-to-pay
Source: PLoS One. 2023 Feb 27;18(2):e0280441. doi: 10.1371/journal.pone.0280441 (PMC9970100; doi:10.1371/journal.pone.0280441)
Supplement: S3 Table — (DOCX) [file pone.0280441.s010.docx]

**S3 Table. Results on questions – Questionnaire Part C.**

| **Results on questions – Questionnaire Part C (excl. questions not relevant, e.g., [8] due to exclusion criteria)** | | |
| --- | --- | --- |
| **Participants' characteristics** | | **Number total** |
|  |  | 380 (100%) |
| Questions about yourself | | |
| [1] Age (in groups) | under 30 years | 0 (0) |
|  | 30 to 34 years | 44 (11.58) |
|  | 36 to 39 years | 24 (6.32) |
|  | 41 to 44 years | 28 (7.37) |
|  | 46 to 49 years | 23 (6.05) |
|  | 51 to 54 years | 46 (12.11) |
|  | 56 to 59 years | 57 (15.00) |
|  | 61 to 64 years | 44 (11.58) |
|  | 66 to 69 years | 31 (8.16) |
|  | 71 to 74 years | 23 (6.05) |
|  | 76 to 79 years | 20 (5.26) |
|  | 81 to 84 years | 15 (3.95) |
|  | 86 to 89 years | 3 (0.79) |
|  | 91 years and older | 1 (0.26) |
|  | no answer / not clear | 21 (5.53) |
| [2] Gender | female | 249 (65.53) |
|  | male | 130 (34.21) |
|  | other | 1 (0.26) |
| [3] Highest educational qualification | (technical) university degree | 166 (43.68) |
|  | vocational training | 115 (30.26) |
|  | (technical) A-level | 22 (5.79) |
|  | high school diploma – 10 years | 26 (6.84) |
|  | high school diploma – 9 years | 13 (3.42) |
|  | no school-leaving qualification | 0 (0) |
|  | other | 3 (0.79) |
|  | no answer / not clear | 35 (9.21) |
| [4] Employment status | full-time | 173 (45.53) |
|  | part-time | 54 (14.21) |
|  | college student (and not employed) | 1 (0.26) |
|  | unemployed | 11 (2.89) |
|  | retirement due to illness | 16 (4.21) |
|  | retirement due to age | 95 (25.00) |
|  | other | 13 (3.42) |
|  | no answer / not clear | 17 (4.47) |
| [5] Monthly (net)household income | under 500 € | 1 (0.26) |
|  | 500 to under 750 € | 7 (1.84) |
|  | 750 to under 1,000 € | 16 (4.21) |
|  | 1,000 to under 1,500 € | 32 (8.42) |
|  | 1,500 to under 2,500 € | 92 (24.21) |
|  | 2,500 to under 3,500 € | 92 (24.21) |
|  | 3,500 to under 4,500 € | 60 (15.79) |
|  | 4,500 to under 5,500 € | 29 (7.63) |
|  | 5,500 to under 6,500 € | 19 (5.00) |
|  | over 6,500 € | 6 (1.58) |
|  | no answer / not clear | 26 (6.84) |
| [6] City or municipality type | large city (> 100,000 inhabitants) | 165 (43.42) |
|  | medium-sized city  (>20,000 –100,000 inhabitants) | 28 (7.37) |
|  | small town  (>5,000 – 20,000 inhabitants) | 42 (11.05) |
|  | rural community / village  (<5,000 inhabitants) | 120 (31.58) |
|  | I do not know | 0 (0) |
|  | no answer / not clear | 25 (6.58) |
| [9] Do you have dental supplementary insurance? | yes | 116 (30.53) |
|  | no | 256 (67.37) |
|  | I do not know | 1 (0.26) |
|  | no answer / not clear | 7 (1.84) |
| [10] Do you have a bonus booklet? | yes | 329 (86.58) |
|  | no | 45 (11.84) |
|  | I do not know | 2 (0.53) |
|  | no answer / not clear | 4 (1.05) |
| If yes: Have you ever submitted a bonus booklet with at least 5 stamps in a row to your health insurance company, so that the coverage is extended by the health insurance company?  *(total: [10] yes)* | yes | 227 (69.00) |
|  | no | 92 (27.96) |
|  | I do not know | 7 (2.13) |
|  | no answer / not clear | 3 (0.91) |
| [11] Would you be eligible for a hardship case application? | yes | 30 (7.89) |
|  | no | 290 (76.32) |
|  | I do not know | 47 (12.37) |
|  | no answer / not clear | 13 (3.42) |
| If yes: Have you ever taken advantage of the hardship case provision for yourself?  *(total: [11] yes)* | yes | 15 (50.00) |
|  | no | 13 (43.33) |
|  | I do not know | 2 (6.67) |
|  | no answer / not clear | 0 (0) |
| Questions about your oral health | | |
| [12] How would you rate your overall oral health? Please rate on a scale from 1 (very good) to 5 (very poor). | 1 (very good) | 34 (8.95) |
|  | 2 (good) | 170 (44.74) |
|  | 3 (average) | 132 (34.74) |
|  | 4 (poor) | 28 (7.37) |
|  | 5 (very poor) | 4 (1.05) |
|  | no answer / not clear | 12 (3.16) |
| [13] Have you ever received a dental crown, e.g., after root canal treatment? | yes | 275 (72.37) |
|  | no | 94 (24.74) |
|  | I do not know | 1 (0.26) |
|  | no answer / not clear | 10 (2.63) |
| [14] Have you ever decided against a dental crown treatment? | yes | 49 (12.89) |
|  | no | 312 (82.11) |
|  | I do not know | 9 (2.37) |
|  | no answer / not clear | 10 (2.63) |
| If yes: What was the reason for deciding against it?  (multiple answers)  *(total: all answers)* | costs too high | 17 (36.17) |
|  | fear of treatment | 3 (6.38) |
|  | no trust in dentist | 8 (17.02) |
|  | I think, it was not necessary | 14 (29.79) |
|  | other | 5 (10.64) |
|  | *total* | 47 (100.00) |
| [15] Change in the law: As of 10/2020, the health insurance company will pay at least 60% of the total costs of the standard treatment instead of the previous 50% (cf. full crown in the non-visible tooth area: new 194.34 €, previously 161.95 € paid by health insurance). Does this change your decision for or against dental crown treatment? | yes | 42 (11.05) |
|  | no | 272 (71.58) |
|  | I do not know | 49 (12.89) |
|  | no answer / not clear | 17 (4.47) |
| If yes: To what extent does your decision change?  *(total: [15] yes)* | I then decide for the standard treatment | 14 (33.33) |
|  | I then decide for a treatment beyond the standard treatment, e.g., a full ceramic crown | 23 (54.76) |
|  | no answer / not clear | 5 (11.90) |
| [16] Does your health status (e.g., constitution, allergies) allow for a dental crown treatment? | yes | 342 (90.00) |
|  | no | 3 (0.79) |
|  | I do not know | 24 (6.32) |
|  | no answer / not clear | 11 (2.89) |
| [17] If dental crowns are strongly visible: Does your decision against a treatment alternative depends on the color (e.g., gold-colored) of the dental crown? | yes | 66 (17.37) |
|  | no, I always decide against a strongly visible dental crown | 238 (62.63) |
|  | I do not know | 61 (16.05) |
|  | no answer / not clear | 15 (3.95) |
| If yes: Which color of dental crown would you choose as treatment alternative?  (multiple answers)  *(total: all answers)* | color golden-metal | 23 (29.87) |
|  | color light grey metallic | 18 (23.38) |
|  | color dark grey metallic | 6 (7.79) |
|  | I do not know | 25 (32.47) |
|  | no answer / not clear | 5 (6.49) |
|  | *total* | 77 (100.00) |
| Assessment of questionnaire | | |
| [18] How difficult was it for you to decide between the treatment alternatives? Please rate on a scale from 1 (very easy) to 5 (very difficult). | 1 (very easy) | 48 (12.63) |
|  | 2 (easy) | 213 (56.05) |
|  | 3 (moderate) | 98 (25.79) |
|  | 4 (difficult) | 7 (1.84) |
|  | 5 (very difficult) | 0 (0) |
|  | no answer / not clear | 14 (3.68) |
| [19] How easy or how difficult did you find it to complete the questionnaire? Please rate using a scale from 1 (very easy) to 5 (very difficult). | 1 (very easy) | 70 (18.42) |
|  | 2 (easy) | 242 (63.68) |
|  | 3 (moderate) | 57 (15.00) |
|  | 4 (difficult) | 4 (1.05) |
|  | 5 (very difficult) | 0 (0) |
|  | no answer / not clear | 7 (1.84) |
| [20] Time for answering questionnaire [in min] | average | 18 min, 28 sec |
|  | min | 4 min |
|  | max | 90 min |
