# Supplementary material for: Patients’ preferences in dental care: A discrete-choice experiment and an analysis of willingness-to-pay
Source: PLoS One. 2023 Feb 27;18(2):e0280441. doi: 10.1371/journal.pone.0280441 (PMC9970100; doi:10.1371/journal.pone.0280441)
Supplement: S4 Table — (DOCX) [file pone.0280441.s011.docx]

**S4 Table. Coefficients of G-MNL estimations, including marginal effects.**

| **Generalized multinomial logit model (G-MNL)** | | | | | | | | |
| --- | --- | --- | --- | --- | --- | --- | --- | --- |
| **Posterior teeth** | | | | | | | | |
| **Attributes (Ref. *negative* levels)** | **Levels** | **Coef.** | **Std. Err.^#^** | **t-value (z)** | **p-value (P>\|z\|)** | **[95% Conf. interval]** | | **Sig.** |
| Aesthetics | *strongly visible* – *reference level* | | | | | | | |
|  | lightly visible | 1.114 | 0.219 | 5.080 | 0.000 | 0.684 | 1.544 | *** |
|  | natural color | 1.530 | 0.306 | 5.000 | 0.000 | 0.930 | 2.129 | *** |
| Compatibility | *1 out of 10,000 people with allergic or local toxic reaction* – *reference level* | | | | | | | |
|  | no risk | 0.680 | 0.157 | 4.320 | 0.000 | 0.371 | 0.988 | *** |
| Durability | *5 years* – *reference level* | | | | | | | |
|  | 10 years | 0.658 | 0.223 | 2.950 | 0.003 | 0.220 | 1.095 | *** |
|  | 15 years | 1.187 | 0.272 | 4.360 | 0.000 | 0.653 | 1.720 | *** |
|  | 25 years | 1.938 | 0.400 | 4.850 | 0.000 | 1.154 | 2.721 | *** |
| Out-of-pocket payment | *600 €* – *reference level* | | | | | | | |
|  | 450 € | 0.498 | 0.133 | 3.750 | 0.000 | 0.237 | 0.758 | *** |
|  | 150 € | 1.492 | 0.300 | 4.970 | 0.000 | 0.904 | 2.079 | *** |
|  | 50 € | 1.403 | 0.276 | 5.090 | 0.000 | 0.863 | 1.943 | *** |
| **Log likelihood** | -2,643.9249 (Iteration 7) | | | | | | | |
| **No. of observations** | 9,039 | | | | | | | |
| **Wald chi2(9)** | 31.48 | | | | | | | |
| **Prob > chi2** | 0.0002 | | | | | | | |
| AIC / BIC (Akaike’s & Schwarz’s Bayesian information criteria): 5,308 / 5,379 | | | | | | | | |
| **Anterior teeth** | | | | | | | | |
| **Attributes (Ref. *negative* levels)** | **Levels** | **Coef.** | **Std. Err.^#^** | **t-value (z)** | **p-value (P>\|z\|)** | **[95% Conf. interval]** | | **Sig.** |
| Aesthetics | *strongly visible* – *reference level* | | | | | | | |
|  | lightly visible | 4.314 | 1.514 | 2.850 | 0.004 | 1.346 | 7.282 | *** |
|  | natural color | 5.949 | 2.041 | 2.910 | 0.004 | 1.948 | 9.950 | *** |
| Compatibility | *1 out of 10,000 people with allergic or local toxic reaction* – *reference level* | | | | | | | |
|  | no risk | 0.392 | 0.186 | 2.100 | 0.035 | 0.027 | 0.758 | ** |
| Durability | *5 years* – *reference level* | | | | | | | |
|  | 10 years | 1.259 | 0.606 | 2.080 | 0.038 | 0.071 | 2.447 | ** |
|  | 15 years | 1.176 | 0.572 | 2.060 | 0.040 | 0.056 | 2.297 | ** |
|  | 25 years | 1.704 | 0.747 | 2.280 | 0.023 | 0.240 | 3.168 | ** |
| Out-of-pocket payment | *600 €* – *reference level* | | | | | | | |
|  | 450 € | -1.261 | 0.473 | -2.670 | 0.008 | -2.188 | -0.335 | *** |
|  | 200 € | 0.617 | 0.287 | 2.150 | 0.032 | 0.054 | 1.180 | ** |
|  | 50 € | 0.060 | 0.286 | 0.210 | 0.833 | -0.499 | 0.620 |  |
| **Log likelihood** | -2,761.8131 (Iteration 15) | | | | | | | |
| **No. of observations** | 9,057 | | | | | | | |
| **Wald chi2(9)** | 11.38 | | | | | | | |
| **Prob > chi2** | 0.2508 | | | | | | | |
| AIC / BIC (Akaike’s & Schwarz’s Bayesian information criteria): 5,544 / 5,615 | | | | | | | | |
| # The sign of the estimated standard deviations is irrelevant: interpret them as being positive. | | | | | | | | |
| *** p<.01, ** p<.05, * p<.1 | | | | | | | | |

| **Generalized multinomial logit model (G-MNL)** | | | | | | | | |
| --- | --- | --- | --- | --- | --- | --- | --- | --- |
| **Average marginal effects, Delta-method** | | | | | | | | |
| **Posterior teeth** | | | | | | | | |
| **Attributes  (Ref. *negative* levels)** | **Levels** | **dy/dx** | **Std. Err.** | **t-value (z)** | **p-value (P>\|z\|)** | **[95% Conf. interval]** | | **Sig.** |
| Aesthetics | lightly visible | 0.557 | 0.110 | 5.080 | 0.000 | 0.342 | 0.772 | *** |
|  | natural color | 1.530 | 0.306 | 5.000 | 0.000 | 0.930 | 2.129 | *** |
| Durability | no risk | 0.680 | 0.157 | 4.320 | 0.000 | 0.371 | 0.988 | *** |
| Compatibility  Durability | 10 years | 0.066 | 0.022 | 2.950 | 0.003 | 0.022 | 0.109 | *** |
|  | 15 years | 0.079 | 0.018 | 4.360 | 0.000 | 0.044 | 0.115 | *** |
|  | 25 years | 0.078 | 0.016 | 4.850 | 0.000 | 0.046 | 0.109 | *** |
| Out-of-pocket payment | 450 € | 0.001 | 0.000 | 3.750 | 0.000 | 0.001 | 0.002 | *** |
|  | 150 € | 0.010 | 0.002 | 4.970 | 0.000 | 0.006 | 0.014 | *** |
|  | 50 € | 0.028 | 0.006 | 5.090 | 0.000 | 0.017 | 0.039 | *** |
| **No. of observations** | 9,039 | | | | | | | |
| **Model VCE** | OIM | | | | | | | |
| **Anterior teeth** | | | | | | | | |
| **Attributes  (Ref. *negative* levels)** | **Levels** | **dy/dx** | **Std. Err.** | **t-value (z)** | **p-value (P>\|z\|)** | **[95% Conf. interval]** | | **Sig.** |
| Aesthetics | lightly visible | 2.157 | 0.769 | 2.810 | 0.005 | 0.650 | 3.664 | *** |
|  | natural color | 5.949 | 2.073 | 2.870 | 0.004 | 1.885 | 10.012 | *** |
| Durability | no risk | 0.392 | 0.187 | 2.100 | 0.036 | 0.026 | 0.759 | ** |
| Compatibility  Durability | 10 years | 0.126 | 0.061 | 2.050 | 0.040 | 0.006 | 0.246 | ** |
|  | 15 years | 0.078 | 0.039 | 2.040 | 0.042 | 0.003 | 0.154 | ** |
|  | 25 years | 0.068 | 0.030 | 2.250 | 0.024 | 0.009 | 0.127 | ** |
| Out-of-pocket payment | 450 € | -0.003 | 0.001 | -2.630 | 0.008 | -0.005 | -0.001 | *** |
|  | 200 € | 0.003 | 0.001 | 2.120 | 0.034 | 0.000 | 0.006 | ** |
|  | 50 € | 0.001 | 0.006 | 0.210 | 0.832 | -0.010 | 0.012 |  |
| **No. of observations** | 9,057 | | | | | | | |
| **Model VCE** | OIM | | | | | | | |
| *** p<.01, ** p<.05, * p<.1 | | | | | | | | |
| Note: dy/dx for factor levels is the discrete change from the base level. | | | | | | | | |
