# Supplementary material for: Patients’ preferences in dental care: A discrete-choice experiment and an analysis of willingness-to-pay
Source: PLoS One. 2023 Feb 27;18(2):e0280441. doi: 10.1371/journal.pone.0280441 (PMC9970100; doi:10.1371/journal.pone.0280441)
Supplement: S5 Table — (DOCX) [file pone.0280441.s012.docx]

**S5 Table. Coefficients of CLM estimations, including marginal effects.**

| **Conditional logit model (CLM)** | | | | | | | | |
| --- | --- | --- | --- | --- | --- | --- | --- | --- |
| **Posterior teeth** | | | | | | | | |
| **Attributes  (Ref. *negative* levels)** | **Levels** | **Coef.** | **Std. Err.** | **t-value (z)** | **p-value (P>\|z\|)** | **[95% Conf. interval]** | | **Sig.** |
| Aesthetics | *strongly visible – reference level* | | | | | | | |
|  | lightly visible | -0.1249 | 0.0371 | -3.37 | 0.001 | -0.198 | -0.052 | *** |
|  | natural color | 0.2575 | 0.0371 | 6.95 | 0.000 | 0.185 | 0.33 | *** |
| Compatibility | *1 out of 10,000 people with allergic or local toxic reaction – reference level* | | | | | | | |
|  | no risk | 0.1136 | 0.0253 | 4.49 | 0.000 | 0.064 | 0.163 | *** |
| Durability | *5 years – reference level* | | | | | | | |
|  | 10 years | -0.501 | 0.0484 | -10.35 | 0.000 | -0.596 | -0.406 | *** |
|  | 15 years | -0.0493 | 0.0461 | -1.07 | 0.284 | -0.14 | 0.041 |  |
|  | 25 years | 0.4972 | 0.0445 | 11.18 | 0.000 | 0.41 | 0.584 | *** |
| Out-of-pocket-payment | *600 € – reference level* | | | | | | | |
|  | 450 € | -0.5139 | 0.0485 | -10.6 | 0.000 | -0.609 | -0.419 | *** |
|  | 150 € | 0.1723 | 0.0453 | 3.81 | 0.000 | 0.084 | 0.261 | *** |
|  | 50 € | 0.1486 | 0.0448 | 3.32 | 0.001 | 0.061 | 0.236 | *** |
| **Log likelihood** | -4,867 (Iteration 2) | | | | | | | |
| **Pseudo R^2** | 0.047 | | | | | | | |
| **No. of observations** | 9,120 | | | | | | | |
| **No. of choices** | 3,040 (n=8 per participant) | | | | | | | |
| AIC / BIC (Akaike’s & Schwarz’s Bayesian information criteria): 9,753 / 9,817 | | | | | | | | |
| **Anterior teeth** | | | | | | | | |
| **Attributes  (Ref. *negative* levels)** | **Levels** | **Coef.** | **Std. Err.** | **t-value (z)** | **p-value (P>\|z\|)** | **[95% Conf. interval]** | | **Sig.** |
| Aesthetics | *strongly visible – reference level* | | | | | | | |
|  | lightly visible | -0.2474 | 0.04 | -6.19 | 0.000 | -0.326 | -0.169 | *** |
|  | natural color | 1.1019 | 0.0407 | 27.09 | 0.000 | 1.022 | 1.182 | *** |
| Compatibility | *1 out of 10,000 people with allergic or local toxic reaction – reference level* | | | | | | | |
|  | no risk | -0.0418 | 0.028 | -1.49 | 0.135 | -0.097 | 0.013 |  |
| Durability | *5 years – reference level* | | | | | | | |
|  | 10 years | -0.3885 | 0.0529 | -7.34 | 0.000 | -0.492 | -0.285 | *** |
|  | 15 years | -0.1791 | 0.0519 | -3.45 | 0.001 | -0.281 | -0.077 | *** |
|  | 25 years | 0.2288 | 0.0499 | 4.59 | 0.000 | 0.131 | 0.326 | *** |
| Out-of-pocket-payment | *600 € – reference level* | | | | | | | |
|  | 450 € | -0.3478 | 0.0531 | -6.54 | 0.000 | -0.452 | -0.244 | *** |
|  | 200 € | -0.0782 | 0.0507 | -1.54 | 0.123 | -0.177 | 0.021 |  |
|  | 50 € | -0.0479 | 0.051 | -0.94 | 0.348 | -0.148 | 0.052 |  |
| **Log likelihood** | -4,608 (Iteration 2) | | | | | | | |
| **Pseudo R^2** | 0.099 | | | | | | | |
| **No. of observations** | 9,120 | | | | | | | |
| **No. of choices** | 3,040 (n=8 per participant) | | | | | | | |
| AIC / BIC (Akaike’s & Schwarz’s Bayesian information criteria): 9,234 / 9,298 | | | | | | | | |
| *** p<.01, ** p<.05, * p<.1 | | | | | | | | |

| **Conditional logit model (CLM)** | | | | | | | | |
| --- | --- | --- | --- | --- | --- | --- | --- | --- |
| **Average marginal effects, Delta-method** | | | | | | | | |
| **Posterior teeth** | | | | | | | | |
| **Attributes  (Ref. *negative* levels)** | **Levels** | **dy/dx** | **Std.Err.** | **t-value (z)** | **p-value (P>\|z\|)** | **[95% Conf. interval]** | | **Sig.** |
| Aesthetics  (Ref. "strongly visible") | lightly visible | 0.041 | 0.006 | 6.580 | 0.000 | 0.029 | 0.053 | *** |
|  | natural color | 0.151 | 0.012 | 12.320 | 0.000 | 0.127 | 0.175 | *** |
| Compatibility  (Ref. "1 out of 10,000 people with allergic or local toxic reaction") | no risk | 0.094 | 0.010 | 9.220 | 0.000 | 0.074 | 0.114 | *** |
| Durability  (Ref. "5 years") | 10 years | -0.000 | 0.001 | -0.220 | 0.824 | -0.003 | 0.003 |  |
|  | 15 years | 0.005 | 0.001 | 5.330 | 0.000 | 0.003 | 0.007 | *** |
|  | 25 years | 0.007 | 0.001 | 13.340 | 0.000 | 0.006 | 0.008 | *** |
| Out-of-pocket-payment  (Ref. "600 €") | 450 € | -0.000 | 0.000 | -2.200 | 0.027 | -0.000 | -0.000 | ** |
|  | 150 € | 0.001 | 0.000 | 6.840 | 0.000 | 0.000 | 0.001 | *** |
|  | 50 € | 0.002 | 0.000 | 7.480 | 0.000 | 0.001 | 0.003 | *** |
| **No. of observations** | 9,120 | | | | | | | |
| **Anterior teeth** | | | | | | | | |
| **Attributes  (Ref. *negative* levels)** | **Levels** | **dy/dx** | **Std.Err.** | **t-value (z)** | **p-value (P>\|z\|)** | **[95%Conf.**  **interval]** | | **Sig.** |
| Aesthetics  (Ref. "strongly visible") | lightly visible | 0.072 | 0.007 | 9.800 | 0.000 | 0.057 | 0.086 | *** |
|  | natural color | 0.427 | 0.013 | 33.150 | 0.000 | 0.402 | 0.452 | *** |
| Compatibility  (Ref. "1 out of 10,000 people with allergic or local toxic reaction") | no risk | 0.001 | 0.012 | 0.090 | 0.931 | -0.023 | 0.025 |  |
| Durability  (Ref. "5 years") | 10 years | -0.012 | 0.002 | -6.920 | 0.000 | -0.015 | -0.008 | *** |
|  | 15 years | -0.005 | 0.001 | -4.400 | 0.000 | -0.007 | -0.003 | *** |
|  | 25 years | 0.000 | 0.001 | 0.280 | 0.780 | -0.001 | 0.001 |  |
| Out-of-pocket-payment  (Ref. "600 €") | 450 € | -0.000 | 0.000 | -8.390 | 0.000 | -0.000 | -0.000 | *** |
|  | 200 € | -0.000 | 0.000 | -5.380 | 0.000 | -0.001 | -0.000 | *** |
|  | 50 € | -0.002 | 0.000 | -4.640 | 0.000 | -0.002 | -0.001 | *** |
| **No. of observations** | 9,120 | | | | | | | |
| *** p<.01, ** p<.05, * p<.1 | | | | | | | | |
| Note: dy/dx for factor levels is the discrete change from the base level. | | | | | | | | |
