# Supplementary material for: Patients’ preferences in dental care: A discrete-choice experiment and an analysis of willingness-to-pay
Source: PLoS One. 2023 Feb 27;18(2):e0280441. doi: 10.1371/journal.pone.0280441 (PMC9970100; doi:10.1371/journal.pone.0280441)
Supplement: S6 Table — (DOCX) [file pone.0280441.s013.docx]

**S6 Table. Coefficients of MXL estimations for anterior teeth.**

| **Mixed logit model (MXL)** | | | | | | | | |
| --- | --- | --- | --- | --- | --- | --- | --- | --- |
| **Anterior teeth** | | | | | | | | |
| **Attributes  (Ref. *negative* levels)** | **Levels** | **Coef.** | **Std. Err.** | **t-value (z)** | **p-value (P>\|z\|)** | **[95% Conf. interval]** | | **Sig.** |
| Aesthetics | *strongly visible* – *reference level* | | | | | | | |
|  | lightly visible | 1.026 | 0.175 | 5.880 | 0.000 | 0.684 | 1.368 | *** |
|  | natural color | 3.392 | 0.174 | 19.520 | 0.000 | 3.052 | 3.733 | *** |
| Compatibility | *1 out of 10,000 people with allergic or local toxic reaction* – *reference level* | | | | | | | |
|  | no risk | 0.200 | 0.093 | 2.160 | 0.031 | 0.019 | 0.381 | ** |
| Durability | *5 years* – *reference level* | | | | | | | |
|  | 10 years | -0.047 | 0.119 | -0.390 | 0.695 | -0.279 | 0.186 |  |
|  | 15 years | -0.103 | 0.123 | -0.840 | 0.402 | -0.343 | 0.138 |  |
|  | 25 years | 0.835 | 0.142 | 5.880 | 0.000 | 0.557 | 1.114 | *** |
| Out-of-pocket payment | *600 €* – *reference level* | | | | | | | |
|  | 450 € | -0.103 | 0.134 | -0.770 | 0.442 | -0.366 | 0.160 |  |
|  | 200 € | 0.157 | 0.113 | 1.390 | 0.164 | -0.064 | 0.379 |  |
|  | 50 € | 0.124 | 0.126 | 0.980 | 0.326 | -0.123 | 0.370 |  |
| **Log likelihood** | -2,326.9783 (Iteration 7) | | | | | | | |
| **Prob > chi2** | 0.0 | | | | | | | |
| **LR chi2(9)** | 753.43 | | | | | | | |
| **No. of observations** | 9,057 | | | | | | | |
| AIC / BIC (Akaike’s & Schwarz’s Bayesian information criteria): 4,692 / 4,827 | | | | | | | | |
| *** p<.01, ** p<.05, * p<.1 | | | | | | | | |
