# Supplementary material for: Patients’ preferences in dental care: A discrete-choice experiment and an analysis of willingness-to-pay
Source: PLoS One. 2023 Feb 27;18(2):e0280441. doi: 10.1371/journal.pone.0280441 (PMC9970100; doi:10.1371/journal.pone.0280441)
Supplement: S7 Table — (DOCX) [file pone.0280441.s014.docx]

**S7 Table. Marginal effects of MXL estimations.**

| **Mixed logit model (MXL)** | | | | | | | | |
| --- | --- | --- | --- | --- | --- | --- | --- | --- |
| **Average marginal effects, Delta-method** | | | | | | | | |
| **Posterior teeth** | | | | | | | | |
| **Attributes  (Ref. *negative* levels)** | **Levels** | **dy/dx** | **Std. Err.** | **t-value (z)** | **p-value (P>\|z\|)** | **[95% Conf. interval]** | | **Sig.** |
| Aesthetics | lightly visible | 0.334 | 0.052 | 6.450 | 0.000 | 0.232 | 0.435 | *** |
|  | natural color | 1.247 | 0.111 | 11.230 | 0.000 | 1.029 | 1.464 | *** |
| Compatibility | no risk | 0.470 | 0.085 | 5.560 | 0.000 | 0.305 | 0.636 | *** |
| Durability | 10 years | 0.016 | 0.011 | 1.510 | 0.132 | -0.005 | 0.038 |  |
|  | 15 years | 0.030 | 0.008 | 3.660 | 0.000 | 0.014 | 0.046 | *** |
|  | 25 years | 0.065 | 0.006 | 11.500 | 0.000 | 0.054 | 0.076 | *** |
| Out-of-pocket payment | 450 € | 0.000 | 0.000 | 1.460 | 0.144 | -0.000 | 0.001 |  |
|  | 150 € | 0.006 | 0.001 | 8.810 | 0.000 | 0.005 | 0.008 | *** |
|  | 50 € | 0.018 | 0.003 | 6.980 | 0.000 | 0.013 | 0.023 | *** |
| **No. of observations** | 9,039 | | | | | | | |
| **Expression** | Linear prediction, predict() | | | | | | | |
| **Model VCE** | OIM | | | | | | | |
| **Anterior teeth** | | | | | | | | |
| **Attributes  (Ref. *negative* levels)** | **Levels** | **dy/dx** | **Std. Err.** | **t-value (z)** | **p-value (P>\|z\|)** | **[95% Conf. interval]** | | **Sig.** |
| Aesthetics | lightly visible | 0.465 | 0.091 | 5.100 | 0.000 | 0.286 | 0.643 | *** |
|  | natural color | 3.449 | 0.177 | 19.500 | 0.000 | 3.102 | 3.796 | *** |
| Compatibility | no risk | 0.242 | 0.092 | 2.630 | 0.009 | 0.062 | 0.422 | *** |
| Durability | 10 years | -0.004 | 0.012 | -0.320 | 0.752 | -0.027 | 0.020 |  |
|  | 15 years | -0.006 | 0.008 | -0.740 | 0.458 | -0.022 | 0.010 |  |
|  | 25 years | 0.036 | 0.006 | 6.380 | 0.000 | 0.025 | 0.047 | *** |
| Out-of-pocket payment | 450 € | -0.000 | 0.000 | -1.260 | 0.209 | -0.001 | 0.000 |  |
|  | 200 € | 0.001 | 0.001 | 1.450 | 0.148 | -0.000 | 0.002 |  |
|  | 50 € | 0.002 | 0.002 | 0.620 | 0.536 | -0.003 | 0.006 |  |
| **No. of observations** | 9,057 | | | | | | | |
| **Expression** | Linear prediction, predict() | | | | | | | |
| **Model VCE** | OIM | | | | | | | |
| *** p<.01, ** p<.05, * p<.1 | | | | | | | | |
| Note: dy/dx for factor levels is the discrete change from the base level. | | | | | | | | |
