# Supplementary material for: Patients’ preferences in dental care: A discrete-choice experiment and an analysis of willingness-to-pay
Source: PLoS One. 2023 Feb 27;18(2):e0280441. doi: 10.1371/journal.pone.0280441 (PMC9970100; doi:10.1371/journal.pone.0280441)
Supplement: S8 Table — (DOCX) [file pone.0280441.s015.docx]

**S8 Table. Coefficients of ASCL estimations "treatment" vs. "no treatment (opt-out)".**

| **Alternative specific constant logit model (ASCL) – Choice of "treatment" or "no treatment (opt-out)"** | | | | | | | |
| --- | --- | --- | --- | --- | --- | --- | --- |
| **Posterior teeth** | | | | | | | |
| **Participants' characteristics** – **Ref. Choice of "treatment"** | **Coef.** | **Std. Err.** | **t-value (z)** | **p-value (P>\|z\|)** | **[95% Conf. interval]** | | **Sig.** |
| Age | 0.119 | 0.024 | 4.93 | 0 | 0.072 | 0.166 | *** |
| *Gender (Ref. level: Female)* | | | | | | | |
| 2. Male | -0.229 | 0.126 | -1.82 | 0.069 | -0.476 | 0.018 | * |
| 3. Other | -13.293 | 595.655 | -0.02 | 0.982 | -1180.755 | 1154.169 |  |
| Income | 0.035 | 0.037 | 0.95 | 0.343 | -0.037 | 0.107 |  |
| *Employment (Ref. level: Full-time employed)* | | | | | | | |
| 2. Not full-time employed or retired | 0.066 | 0.134 | 0.50 | 0.62 | -0.196 | 0.329 |  |
| Education | 0.025 | 0.048 | 0.52 | 0.601 | -0.069 | 0.119 |  |
| Rural / urban region | 0.131 | 0.039 | 3.38 | 0.001 | 0.055 | 0.207 | *** |
| *Importance of attributes* | | | | | | | |
| Aesthetics | 0.154 | 0.054 | 2.84 | 0.004 | 0.048 | 0.26 | *** |
| Compatibility | 0.075 | 0.094 | 0.80 | 0.425 | -0.11 | 0.261 |  |
| Durability | 0.018 | 0.115 | 0.15 | 0.879 | -0.208 | 0.243 |  |
| Out-of-pocket payment | 0.083 | 0.061 | 1.38 | 0.169 | -0.035 | 0.202 |  |
| *Incentive measures* | | | | | | | |
| Bonus booklet | 0.01 | 0.006 | 1.65 | 0.099 | -0.002 | 0.021 | * |
| Supplementary insurance | 0 | 0.011 | -0.01 | 0.991 | -0.022 | 0.021 |  |
| Combination of bonus booklet & suppl. insurance | 0.335 | 0.114 | 2.94 | 0.003 | 0.112 | 0.558 | *** |
| **Log likelihood** | -1,116.3966 (Iteration 8) | | | | | | |
| **Prob > chi2** | 0.0 | | | | | | |
| **Wald chi2(14)** | 76.83 | | | | | | |
| **No. of observations (in model)** | 4,058 | | | | | | |
| **No. of cases (in model)** | 2,029 | | | | | | |
| **No. of choices** | 3,013 | | | | | | |
| **Choice of** **"opt-out"** | 774 (25.7%) | | | | | | |
| AIC / BIC (Akaike’s & Schwarz’s Bayesian information criteria): 2,263 / 2,357 | | | | | | | |
| **Anterior teeth** | | | | | | | |
| **Participants' characteristics** – **Ref. Choice of "treatment"** | **Coef.** | **Std. Err.** | **t-value (z)** | **p-value (P>\|z\|)** | **[95% Conf. interval]** | | **Sig.** |
| Age | 0.087 | 0.022 | 4.06 | 0 | 0.045 | 0.13 | *** |
| *Gender (Ref. level: Female)* | | | | | | | |
| 2. Male | -0.322 | 0.114 | -2.82 | 0.005 | -0.545 | -0.098 | *** |
| 3. Other | -14.532 | 955.906 | -0.02 | 0.988 | -1888.073 | 1859.009 |  |
| Income | 0.077 | 0.033 | 2.34 | 0.019 | 0.012 | 0.142 | ** |
| *Employment (Ref. level: Full-time employed)* | | | | | | | |
| 2. Not full-time employed or retired | 0.18 | 0.121 | 1.49 | 0.137 | -0.057 | 0.417 |  |
| Education | -0.046 | 0.044 | -1.04 | 0.298 | -0.134 | 0.041 |  |
| Rural / urban region | 0.129 | 0.036 | 3.63 | 0 | 0.059 | 0.198 | *** |
| *Importance of attributes* | | | | | | | |
| Aesthetics | 0.1 | 0.049 | 2.05 | 0.04 | 0.004 | 0.195 | ** |
| Compatibility | 0.13 | 0.085 | 1.53 | 0.125 | -0.036 | 0.297 |  |
| Durability | -0.004 | 0.105 | -0.04 | 0.969 | -0.211 | 0.202 |  |
| Out-of-pocket payment | 0.137 | 0.055 | 2.51 | 0.012 | 0.03 | 0.245 | ** |
| *Incentive measures* | | | | | | | |
| Bonus booklet | 0.006 | 0.006 | 10.09 | 0.276 | -0.005 | 0.017 |  |
| Supplementary insurance | 0.012 | 0.008 | 1.58 | 0.115 | -0.003 | 0.027 |  |
| Combination of bonus booklet & suppl. insurance | 0.377 | 0.105 | 3.60 | 0 | 0.172 | 0.582 | *** |
| **Log likelihood** | -1,286.4282 (Iteration 9) | | | | | | |
| **Prob > chi2** | 0.0 | | | | | | |
| **Wald chi2(14)** | 87.77 | | | | | | |
| **No. of observations (in model)** | 4,054 | | | | | | |
| **No. of cases (in model)** | 2,027 | | | | | | |
| **No. of choices** | 3,019 | | | | | | |
| **Choice of "opt-out"** | 1,122 (37.2%) | | | | | | |
| AIC / BIC (Akaike’s & Schwarz’s Bayesian information criteria): 2,603 / 2,697 | | | | | | | |
| *** p<.01, ** p<.05, * p<.1 | | | | | | | |
