# Supplementary material for: Patients’ preferences in dental care: A discrete-choice experiment and an analysis of willingness-to-pay
Source: PLoS One. 2023 Feb 27;18(2):e0280441. doi: 10.1371/journal.pone.0280441 (PMC9970100; doi:10.1371/journal.pone.0280441)
Supplement: S9 Table — (DOCX) [file pone.0280441.s016.docx]

**S9 Table. Coefficients of ASCL estimations "SHI standard care" and "treatment beyond SHI standard care (SHI+)".**

| **Alternative specific constant logit model (ASCL) – Choice of "SHI standard care" or "treatment above SHI standard care (SHI+)"** | | | | | | | |
| --- | --- | --- | --- | --- | --- | --- | --- |
| **Posterior teeth** | | | | | | | |
| **Participants' characteristics** – **Ref. Choice of "SHI standard care"** | **Coef.** | **Std. Err.** | **t-value (z)** | **p-value (P>\|z\|)** | **[95% Conf. interval]** | | **Sig.** |
| Age | 0.022 | 0.031 | 0.70 | 0.486 | -0.039 | 0.083 |  |
| *Gender (Ref. level: Female)* | | | | | | | |
| 2. Male | 0.082 | 0.163 | 0.50 | 0.618 | -0.239 | 0.402 |  |
| 3. Other | -1.871 | 1.153 | -1.62 | 0.105 | -4.131 | 0.39 |  |
| Income | 0.004 | 0.049 | 0.09 | 0.93 | -0.092 | 0.101 |  |
| *Employment (Ref. level: Full-time employed)* | | | | | | | |
| 2. Not full-time employed or retired | 0.218 | 0.182 | 1.20 | 0.231 | -0.138 | 0.573 |  |
| Education | 0.042 | 0.066 | 0.64 | 0.52 | -0.086 | 0.171 |  |
| Rural / urban region | -0.031 | 0.052 | -0.60 | 0.549 | -0.134 | 0.071 |  |
| *Importance of attributes* | | | | | | | |
| Aesthetics | -0.243 | 0.073 | -3.34 | 0.001 | -0.385 | -0.1 | *** |
| Compatibility | 0.152 | 0.121 | 1.26 | 0.209 | -0.085 | 0.39 |  |
| Durability | -0.208 | 0.154 | -1.36 | 0.175 | -0.51 | 0.093 |  |
| Out-of-pocket payment | 0.105 | 0.079 | 1.32 | 0.185 | -0.05 | 0.26 |  |
| *Incentive measures* | | | | | | | |
| Bonus booklet | -0.008 | 0.007 | -1.14 | 0.256 | -0.022 | 0.006 |  |
| Supplementary insurance | 0.002 | 0.012 | 0.20 | 0.838 | -0.021 | 0.026 |  |
| Combination of bonus booklet & suppl. insurance | -0.057 | 0.156 | -0.37 | 0.715 | -0.362 | 0.248 |  |
| **Log likelihood** | -626.67361 (Iteration 4) | | | | | | |
| **Prob > chi2** | 0.0112 | | | | | | |
| **Wald chi2(14)** | 28.78 | | | | | | |
| **No. of observations (in model)** | 2,086 | | | | | | |
| **No. of cases (in model)** | 1,043 | | | | | | |
| **No. of choices** | 1,533 | | | | | | |
| **Choice of "SHI+"** | 480 (31,3%) | | | | | | |
| AIC / BIC (Akaike’s & Schwarz’s Bayesian information criteria): 1,283 / 1,368 | | | | | | | |
| **Anterior teeth** | | | | | | | |
| **Participants' characteristics** – **Ref. Choice of** **"SHI standard care"** | **Coef.** | **Std. Err.** | **t-value (z)** | **p-value (P>\|z\|)** | **[95% Conf. interval]** | | **Sig.** |
| Age | 0.048 | 0.038 | 1.28 | 0.201 | -0.026 | 0.123 |  |
| *Gender (Ref. level: Female)* | | | | | | | |
| 2. Male | -0.195 | 0.196 | -1.00 | 0.319 | -0.579 | 0.189 |  |
| 3. Other | -12.806 | 517.522 | -0.02 | 0.98 | -1027.13 | 1001.518 |  |
| Income | -0.034 | 0.06 | -0.58 | 0.565 | -0.151 | 0.083 |  |
| *Employment (Ref. level: Full-time employed)* | | | | | | | |
| 2. Not full-time employed or retired | 0.132 | 0.217 | 0.61 | 0.543 | -0.294 | 0.558 |  |
| Education | 0.058 | 0.077 | 0.76 | 0.448 | -0.092 | 0.209 |  |
| Rural / urban region | 0.065 | 0.063 | 1.03 | 0.301 | -0.058 | 0.188 |  |
| *Importance of attributes* | | | | | | | |
| Aesthetics | -0.18 | 0.087 | -2.06 | 0.04 | -0.351 | -0.009 | ** |
| Compatibility | 0.197 | 0.145 | 1.36 | 0.173 | -0.087 | 0.482 |  |
| Durability | -0.159 | 0.183 | -0.87 | 0.385 | -0.518 | 0.2 |  |
| Out-of-pocket payment | 0.17 | 0.096 | 1.76 | 0.078 | -0.019 | 0.359 | * |
| *Incentive measures* | | | | | | | |
| Bonus booklet | 0.019 | 0.011 | 1.64 | 0.1 | -0.004 | 0.041 |  |
| Supplementary insurance | 0.012 | 0.013 | 0.92 | 0.358 | -0.014 | 0.037 |  |
| Combination of bonus booklet & suppl. insurance | -0.026 | 0.188 | -0.14 | 0.891 | -0.394 | 0.342 |  |
| **Log likelihood** | -411.3787 (Iteration 8) | | | | | | |
| **Prob > chi2** | 0.1060 | | | | | | |
| **Wald chi2(14)** | 20.83 | | | | | | |
| **No. of observations (in model)** | 1,224 | | | | | | |
| **No. of cases (in model)** | 612 | | | | | | |
| **No. of choices** | 902 | | | | | | |
| **Choice of "SHI+"** | 449 (49,8%) | | | | | | |
| AIC / BIC (Akaike’s & Schwarz’s Bayesian information criteria): 853 / 929 | | | | | | | |
| *** p<.01, ** p<.05, * p<.1 | | | | | | | |

Further abbreviation:

SHI+ – treatment presenting attribute levels beyond statutory health insurance (SHI) standard care
