# Supplementary material for: Patients’ preferences in dental care: A discrete-choice experiment and an analysis of willingness-to-pay
Source: PLoS One. 2023 Feb 27;18(2):e0280441. doi: 10.1371/journal.pone.0280441 (PMC9970100; doi:10.1371/journal.pone.0280441)
Supplement: S10 Table — (DOCX) [file pone.0280441.s017.docx]

**S10 Table. WTP analysis framework, and results.**

| **Attributes & consideration /**  **teeth area & level expression** | **Aesthetics** | | **Compatibility** | | **Durability** | | **Out-of-pocket payment** |
| --- | --- | --- | --- | --- | --- | --- | --- |
|  | **Level** | **WTP value** | **Level** | **WTP value** | **Level** | **WTP value** |  |
| **Posterior teeth** | | | | | | | |
| Positive ("highest quality") | natural color | 379.52 € | no risk | 162.17 € | 25 years | 508.30 € | 50 € |
|  | lightly visible |  | risk | -297.46 €† | 15 years | 258.06 € | 150 € |
|  | strongly visible | -672.82 €† |  | | 10 years |  | 450 € |
| Negative |  | |  | | 5 years |  | 600 € |
| **Anterior teeth** | | | | | | | |
| Positive ("highest quality") | natural color | 914.30 € | no risk | 92.37 € | 25 years | 282.20 € | 50 € |
|  | lightly visible | 362.22 € | risk | -513.33 €† | 15 years |  | 200 € |
|  | strongly visible |  |  | | 10 years | 72.89 € | 450 € |
| Negative |  | |  | | 5 years |  | 600 € |
| *Abbreviation: WTP – willingness-to-pay, †estimation with positive reference level (others: with negative reference level)*  *Legend: statutory health insurance (SHI) standard care* | | | | | | | |
