# Supplementary material for: Patients’ preferences in dental care: A discrete-choice experiment and an analysis of willingness-to-pay
Source: PLoS One. 2023 Feb 27;18(2):e0280441. doi: 10.1371/journal.pone.0280441 (PMC9970100; doi:10.1371/journal.pone.0280441)
Supplement: S11 Table — (DOCX) [file pone.0280441.s018.docx]

**S11 Table. Tables on correlation analysis, VIF values, and regression analysis.**

**(I) Analysis "Individual WTPmax"**

**I.1 Correlation analysis, VIF values, and regression analysis for posterior teeth (PT)**

**I.1.1 Correlation analysis – PT**

| **Variables** | **(1)** | **(2)** | **(3)** | **(4)** | **(5)** | **(6)** | **(7)** | **(8)** | **(9)** | **(10)** | **(11)** | **(12)** | **(13)** | **(14)** | **(15)** | **(16)** | **(17)** |
| --- | --- | --- | --- | --- | --- | --- | --- | --- | --- | --- | --- | --- | --- | --- | --- | --- | --- |
| (1) WTPmax (PT) | 1.000 |  |  |  |  |  |  |  |  |  |  |  |  |  |  |  |  |
|  |  |  |  |  |  |  |  |  |  |  |  |  |  |  |  |  |  |
| (2) Age | -0.104* | 1.000 |  |  |  |  |  |  |  |  |  |  |  |  |  |  |  |
|  | (0.049) |  |  |  |  |  |  |  |  |  |  |  |  |  |  |  |  |
| Gender | | | | | | | | | | | | | | | | | |
| (3) Female | -0.033 | -0.166* | 1.000 |  |  |  |  |  |  |  |  |  |  |  |  |  |  |
|  | (0.526) | (0.002) |  |  |  |  |  |  |  |  |  |  |  |  |  |  |  |
| (4) Male | 0.028 | 0.167* | -0.994* | 1.000 |  |  |  |  |  |  |  |  |  |  |  |  |  |
|  | (0.580) | (0.001) | (0.000) |  |  |  |  |  |  |  |  |  |  |  |  |  |  |
| (5) Other (gender) | 0.039 | -0.011 | -0.071 | -0.037 | 1.000 |  |  |  |  |  |  |  |  |  |  |  |  |
|  | (0.452) | (0.832) | (0.168) | (0.472) |  |  |  |  |  |  |  |  |  |  |  |  |  |
| (6) Income | 0.117* | -0.169* | -0.117* | 0.121* | -0.029 | 1.000 |  |  |  |  |  |  |  |  |  |  |  |
|  | (0.027) | (0.002) | (0.028) | (0.023) | (0.585) |  |  |  |  |  |  |  |  |  |  |  |  |
| Employment | | | | | | | | | | | | | | | | | |
| (7) Full time employed | 0.013 | -0.537* | -0.074 | 0.068 | 0.054 | 0.334* | 1.000 |  |  |  |  |  |  |  |  |  |  |
|  | (0.813) | (0.000) | (0.165) | (0.201) | (0.312) | (0.000) |  |  |  |  |  |  |  |  |  |  |  |
| (8) Not full time employed or retired | -0.013 | 0.537* | 0.074 | -0.068 | -0.054 | -0.334* | -1.000 | 1.000 |  |  |  |  |  |  |  |  |  |
|  | (0.813) | (0.000) | (0.165) | (0.201) | (0.312) | (0.000) | (1.000) |  |  |  |  |  |  |  |  |  |  |
| (9) Education | -0.028 | 0.015 | 0.019 | -0.026 | 0.058 | -0.232* | -0.005 | 0.005 | 1.000 |  |  |  |  |  |  |  |  |
|  | (0.600) | (0.784) | (0.725) | (0.635) | (0.287) | (0.000) | (0.931) | (0.931) |  |  |  |  |  |  |  |  |  |
| (10) Rural / urban region | -0.005 | 0.090 | -0.111* | 0.105* | 0.052 | 0.046 | -0.044 | 0.044 | -0.029 | 1.000 |  |  |  |  |  |  |  |
|  | (0.929) | (0.101) | (0.037) | (0.048) | (0.326) | (0.402) | (0.433) | (0.433) | (0.603) |  |  |  |  |  |  |  |  |
| Importance of attributes | | | | | | | | | | | | | | | | | |
| (11) Aesthetics | 0.082 | -0.054 | 0.144* | -0.144* | -0.006 | 0.041 | 0.054 | -0.054 | 0.002 | 0.083 | 1.000 |  |  |  |  |  |  |
|  | (0.113) | (0.314) | (0.005) | (0.005) | (0.914) | (0.443) | (0.313) | (0.313) | (0.971) | (0.121) |  |  |  |  |  |  |  |
| (12) Compatibility | 0.004 | 0.008 | 0.164* | -0.153* | -0.107* | 0.029 | 0.020 | -0.020 | -0.057 | 0.043 | 0.329* | 1.000 |  |  |  |  |  |
|  | (0.946) | (0.886) | (0.001) | (0.003) | (0.039) | (0.595) | (0.711) | (0.711) | (0.299) | (0.427) | (0.000) |  |  |  |  |  |  |
| (13) Duration | 0.027 | -0.098 | 0.063 | -0.067 | 0.030 | 0.093 | 0.102 | -0.102 | -0.067 | 0.000 | 0.133* | 0.382* | 1.000 |  |  |  |  |
|  | (0.599) | (0.065) | (0.222) | (0.199) | (0.567) | (0.083) | (0.058) | (0.058) | (0.222) | (1.000) | (0.010) | (0.000) |  |  |  |  |  |
| (14) Out-of-pocket payment | -0.109* | -0.009 | 0.124* | -0.120* | -0.034 | -0.243* | -0.054 | 0.054 | 0.110* | -0.062 | 0.024 | 0.035 | 0.116* | 1.000 |  |  |  |
|  | (0.035) | (0.872) | (0.017) | (0.020) | (0.509) | (0.000) | (0.318) | (0.318) | (0.044) | (0.251) | (0.645) | (0.498) | (0.025) |  |  |  |  |
| Incentive measures | | | | | | | | | | | | | | | | | |
| (15) Bonus booklet | 0.090 | 0.242* | 0.129* | -0.132* | 0.019 | -0.016 | -0.067 | 0.067 | 0.004 | -0.112* | 0.038 | 0.093 | -0.035 | 0.013 | 1.000 |  |  |
|  | (0.081) | (0.000) | (0.012) | (0.011) | (0.712) | (0.769) | (0.215) | (0.215) | (0.939) | (0.036) | (0.465) | (0.074) | (0.505) | (0.807) |  |  |  |
| (16) Supplementary insurance | 0.059 | 0.002 | 0.112* | -0.109* | -0.035 | 0.014 | -0.019 | 0.019 | 0.073 | 0.044 | 0.127* | 0.086 | 0.052 | 0.008 | 0.161* | 1.000 |  |
|  | (0.259) | (0.970) | (0.030) | (0.036) | (0.502) | (0.799) | (0.729) | (0.729) | (0.184) | (0.408) | (0.015) | (0.101) | (0.323) | (0.873) | (0.002) |  |  |
| (17) Combination of bonus booklet & suppl. insurance | 0.050 | 0.028 | 0.113* | -0.109* | -0.033 | 0.012 | -0.011 | 0.011 | 0.068 | 0.026 | 0.119* | 0.112* | 0.037 | 0.005 | 0.240* | 0.969* | 1.000 |
|  | (0.335) | (0.596) | (0.028) | (0.033) | (0.521) | (0.816) | (0.837) | (0.837) | (0.213) | (0.631) | (0.022) | (0.030) | (0.474) | (0.920) | (0.000) | (0.000) |  |
| *Abbreviations: WTPmax – highest level value of the attribute out-of-pocket payment for a chosen treatment alternative across all alternatives per participant presenting maximum individual willingness-to-pay (WTP);  *** p<0.01, ** p<0.05, * p<0.1*  *Legend: statistically significant results regarding dependent variable, assumed multicollinearity (one variable to be excluded)* | | | | | | | | | | | | | | | | | |

**I.1.2 VIF - PT**

| **Variable** | **VIF** | **1/VIF** |
| --- | --- | --- |
| Age | 1.698 | .589 |
| Gender | | |
| Male | 1.226 | .816 |
| Other (gender) | 1.069 | .936 |
| Income | 1.275 | .784 |
| Employment | | |
| Full time employed | 1.672 | .598 |
| Education | 1.073 | .932 |
| Rural / urban region | 1.086 | .921 |
| Importance of attributes | | |
| Aesthetics | 1.213 | .824 |
| Compatibility | 1.478 | .677 |
| Duration | 1.293 | .773 |
| Out-of-pocket payment | 1.165 | .858 |
| Incentive measures | | |
| Bonus booklet | 1.294 | .773 |
| Supplementary insurance | 20.816 | .048 |
| Combination of bonus booklet & suppl. insurance | 21.349 | .047 |
| Mean VIF | 4.122 | . |
| Abbreviations: VIF *–* variance inflation factor  Legend: *proof of multicollinearity (one variable to be excluded)* | | |

**I.1.3 Regression analysis – PT**

| **Variables** | **Coef.** | **Std. Err.** | **t-value** | **p-value** | **[95% Conf. interval]** | | | **Sig.** |
| --- | --- | --- | --- | --- | --- | --- | --- | --- |
| Age | -13.783 | 4.97 | -2.77 | .006 | -23.574 | | -3.991 | *** |
| Gender | | | | | | | | |
| Female | -37.832 | 25.746 | -1.47 | .143 | -88.551 | | 12.887 |  |
| Other (gender) | 89.357 | 176.137 | 0.51 | .612 | -257.63 | | 436.343 |  |
| Income | 10.19 | 7.453 | 1.37 | .173 | -4.494 | | 24.873 |  |
| Employment | | | | | | | | |
| Full time employed | -41.869 | 27.792 | -1.51 | .133 | -96.62 | | 12.882 |  |
| Education | -2.769 | 10.019 | -0.28 | .783 | -22.506 | | 16.969 |  |
| Rural / urban region | 8.632 | 8.206 | 1.05 | .294 | -7.534 | | 24.799 |  |
| Importance of attributes | | | | | | | | |
| Aesthetics | 11.699 | 11.009 | 1.06 | .289 | -9.989 | | 33.387 |  |
| Compatibility | -7.26 | 19.164 | -0.38 | .705 | -45.013 | | 30.492 |  |
| Duration | 18.071 | 23.737 | 0.76 | .447 | -28.691 | | 64.833 |  |
| Out-of-pocket payment | -11.702 | 12.223 | -0.96 | .339 | -35.781 | | 12.377 |  |
| Incentive measures | | | | | | | | |
| Bonus booklet | 114.787 | 35.717 | 3.21 | .001 | 44.426 | | 185.149 | *** |
| Supplementary insurance | 7.385 | 24.335 | 0.30 | .762 | -40.555 | | 55.324 |  |
| Constant | 374.685 | 134.149 | 2.79 | .006 | 110.414 | | 638.956 | *** |
| Mean dependent var | 464.881 | | SD dependent var | | | 174.371 | | |
| R-squared | 0.094 | | Number of obs. | | | 252 | | |
| F-test | 1.890 | | Prob > F | | | 0.032 | | |
| Akaike crit. (AIC) | 3,318.621 | | Bayesian crit. (BIC) | | | 3,368.033 | | |
| *Abbreviations: *** p<.01, ** p<.05, * p<.1*  *Legend: statistically significant results* | | | | | | | | |

**I.2 Correlation analysis, VIF values, and regression analysis for anterior teeth (AT)**

**I.2.1 Correlation analysis – AT**

| **Variables** | **(1)** | **(2)** | **(3)** | **(4)** | **(5)** | **(6)** | **(7)** | **(8)** | **(9)** | **(10)** | **(11)** | **(12)** | **(13)** | **(14)** | **(15)** | **(16)** | **(17)** |
| --- | --- | --- | --- | --- | --- | --- | --- | --- | --- | --- | --- | --- | --- | --- | --- | --- | --- |
| (1) WTPmax (AT) | 1.000 |  |  |  |  |  |  |  |  |  |  |  |  |  |  |  |  |
|  |  |  |  |  |  |  |  |  |  |  |  |  |  |  |  |  |  |
| (2) Age | -0.125* | 1.000 |  |  |  |  |  |  |  |  |  |  |  |  |  |  |  |
|  | (0.018) |  |  |  |  |  |  |  |  |  |  |  |  |  |  |  |  |
| Gender | | | | | | | | | | | | | | | | | |
| (3) Female | -0.051 | -0.166* | 1.000 |  |  |  |  |  |  |  |  |  |  |  |  |  |  |
|  | (0.317) | (0.002) |  |  |  |  |  |  |  |  |  |  |  |  |  |  |  |
| (4) Male | 0.047 | 0.167* | -0.994* | 1.000 |  |  |  |  |  |  |  |  |  |  |  |  |  |
|  | (0.356) | (0.001) | (0.000) |  |  |  |  |  |  |  |  |  |  |  |  |  |  |
| (5) Other (gender) | 0.038 | -0.011 | -0.071 | -0.037 | 1.000 |  |  |  |  |  |  |  |  |  |  |  |  |
|  | (0.460) | (0.832) | (0.168) | (0.472) |  |  |  |  |  |  |  |  |  |  |  |  |  |
| (6) Income | 0.129* | -0.169* | -0.117* | 0.121* | -0.029 | 1.000 |  |  |  |  |  |  |  |  |  |  |  |
|  | (0.015) | (0.002) | (0.028) | (0.023) | (0.585) |  |  |  |  |  |  |  |  |  |  |  |  |
| Employment | | | | | | | | | | | | | | | | | |
| (7) Full time employed | 0.086 | -0.537* | -0.074 | 0.068 | 0.054 | 0.334* | 1.000 |  |  |  |  |  |  |  |  |  |  |
|  | (0.108) | (0.000) | (0.165) | (0.201) | (0.312) | (0.000) |  |  |  |  |  |  |  |  |  |  |  |
| (8) Not full time employed or retired | -0.086 | 0.537* | 0.074 | -0.068 | -0.054 | -0.334* | -1.000 | 1.000 |  |  |  |  |  |  |  |  |  |
|  | (0.108) | (0.000) | (0.165) | (0.201) | (0.312) | (0.000) | (1.000) |  |  |  |  |  |  |  |  |  |  |
| (9) Education | 0.002 | 0.015 | 0.019 | -0.026 | 0.058 | -0.232* | -0.005 | 0.005 | 1.000 |  |  |  |  |  |  |  |  |
|  | (0.977) | (0.784) | (0.725) | (0.635) | (0.287) | (0.000) | (0.931) | (0.931) |  |  |  |  |  |  |  |  |  |
| (10) Rural / urban region | -0.014 | 0.090 | -0.111* | 0.105* | 0.052 | 0.046 | -0.044 | 0.044 | -0.029 | 1.000 |  |  |  |  |  |  |  |
|  | (0.793) | (0.101) | (0.037) | (0.048) | (0.326) | (0.402) | (0.433) | (0.433) | (0.603) |  |  |  |  |  |  |  |  |
| Importance of attributes | | | | | | | | | | | | | | | | | |
| (11) Aesthetics | 0.028 | -0.180* | 0.263* | -0.267* | 0.025 | 0.129* | 0.123* | -0.123* | -0.067 | -0.066 | 1.000 |  |  |  |  |  |  |
|  | (0.585) | (0.001) | (0.000) | (0.000) | (0.631) | (0.016) | (0.021) | (0.021) | (0.216) | (0.216) |  |  |  |  |  |  |  |
| (12) Compatibility | -0.043 | 0.038 | 0.213* | -0.217* | 0.039 | -0.027 | -0.044 | 0.044 | 0.016 | -0.056 | 0.288* | 1.000 |  |  |  |  |  |
|  | (0.408) | (0.474) | (0.000) | (0.000) | (0.455) | (0.608) | (0.418) | (0.418) | (0.770) | (0.290) | (0.000) |  |  |  |  |  |  |
| (13) Duration | 0.002 | 0.027 | 0.211* | -0.215* | 0.032 | 0.010 | -0.019 | 0.019 | -0.098 | -0.011 | 0.276* | 0.477* | 1.000 |  |  |  |  |
|  | (0.964) | (0.612) | (0.000) | (0.000) | (0.530) | (0.859) | (0.728) | (0.728) | (0.071) | (0.837) | (0.000) | (0.000) |  |  |  |  |  |
| (14) Out-of-pocket payment | -0.107* | -0.027 | 0.138* | -0.145* | 0.066 | -0.304* | -0.012 | 0.012 | 0.179* | 0.043 | 0.135* | 0.234* | 0.236* | 1.000 |  |  |  |
|  | (0.038) | (0.618) | (0.007) | (0.005) | (0.203) | (0.000) | (0.823) | (0.823) | (0.001) | (0.417) | (0.009) | (0.000) | (0.000) |  |  |  |  |
| Incentive measures | | | | | | | | | | | | | | | | | |
| (15) Bonus booklet | 0.091 | 0.242* | 0.129* | -0.132* | 0.019 | -0.016 | -0.067 | 0.067 | 0.004 | -0.112* | 0.075 | 0.139* | 0.027 | -0.037 | 1.000 |  |  |
|  | (0.079) | (0.000) | (0.012) | (0.011) | (0.712) | (0.769) | (0.215) | (0.215) | (0.939) | (0.036) | (0.151) | (0.007) | (0.600) | (0.481) |  |  |  |
| (16) Supplementary insurance | 0.029 | 0.002 | 0.112* | -0.109* | -0.035 | 0.014 | -0.019 | 0.019 | 0.073 | 0.044 | 0.145* | 0.173* | 0.161* | 0.034 | 0.161* | 1.000 |  |
|  | (0.572) | (0.970) | (0.030) | (0.036) | (0.502) | (0.799) | (0.729) | (0.729) | (0.184) | (0.408) | (0.005) | (0.001) | (0.002) | (0.516) | (0.002) |  |  |
| (17) Combination of bonus booklet & suppl. insurance | 0.028 | 0.028 | 0.113* | -0.109* | -0.033 | 0.012 | -0.011 | 0.011 | 0.068 | 0.026 | 0.131* | 0.200* | 0.141* | 0.025 | 0.240* | 0.969* | 1.000 |
|  | (0.588) | (0.596) | (0.028) | (0.033) | (0.521) | (0.816) | (0.837) | (0.837) | (0.213) | (0.631) | (0.011) | (0.000) | (0.006) | (0.623) | (0.000) | (0.000) |  |
| *Abbreviations: WTPmax – highest level value of the attribute out-of-pocket payment for a chosen treatment alternative across all alternatives per participant presenting maximum individual willingness-to-pay (WTP);  *** p<0.01, ** p<0.05, * p<0.1*  *Legend: statistically significant results regarding dependent variable, assumed multicollinearity (one variable to be excluded)* | | | | | | | | | | | | | | | | | |

**I.2.2 VIF – AT**

| **Variable** | **VIF** | **1/VIF** |
| --- | --- | --- |
| Age | 1.689 | .592 |
| Gender | | |
| Female | 1.27 | .787 |
| Other (gender) | 1.041 | .961 |
| Income | 1.327 | .753 |
| Employment | | |
| Full time employed | 1.636 | .611 |
| Education | 1.098 | .911 |
| Rural / urban region | 1.066 | .938 |
| Importance of attributes | | |
| Aesthetics | 1.302 | .768 |
| Compatibility | 1.499 | .667 |
| Duration | 1.383 | .723 |
| Out-of-pocket payment | 1.334 | .75 |
| Incentive measures | | |
| Bonus booklet | 1.308 | .765 |
| Supplementary insurance | 21.435 | .047 |
| Combination of bonus booklet & suppl. insurance | 22.045 | .045 |
| Mean VIF | 4.245 | . |
| *Abbreviations: VIF – variance inflation factor*  *Legend: proof of multicollinearity (one variable to be excluded)* | | |

**I.6 Regression analysis – AT**

| **Variables** | **Coef.** | **Std. Err.** | **t-value** | **p-value** | **[95% Conf. interval]** | | | **Sig.** |
| --- | --- | --- | --- | --- | --- | --- | --- | --- |
| Age | -13.726 | 5.406 | -2.54 | .012 | -24.375 | | -3.077 | ** |
| Gender | | | | | | | | |
| Female | -91.002 | 28.507 | -3.19 | .002 | -147.158 | | -34.847 | *** |
| Other (gender) | 86.31 | 190.965 | 0.45 | .652 | -289.863 | | 462.483 |  |
| Income | 6.118 | 8.321 | 0.74 | .463 | -10.272 | | 22.509 |  |
| Employment | | | | | | | | |
| Full time employed | -18.398 | 29.949 | -0.61 | .54 | -77.393 | | 40.597 |  |
| Education | 1.328 | 11.072 | 0.12 | .905 | -20.482 | | 23.139 |  |
| Rural / urban region | 4.463 | 8.881 | 0.50 | .616 | -13.032 | | 21.958 |  |
| Importance of attributes | | | | | | | | |
| Aesthetics | 12.455 | 21.865 | 0.57 | .569 | -30.616 | | 55.527 |  |
| Compatibility | -19.114 | 21.152 | -0.90 | .367 | -60.781 | | 22.552 |  |
| Duration | 43.549 | 27.006 | 1.61 | .108 | -9.649 | | 96.747 |  |
| Out-of-pocket payment | -19.555 | 13.13 | -1.49 | .138 | -45.419 | | 6.309 |  |
| Incentive measures | | | | | | | | |
| Bonus booklet | 111.658 | 39.407 | 2.83 | .005 | 34.033 | | 189.284 | *** |
| Supplementary insurance | 2.643 | 26.609 | 0.10 | .921 | -49.774 | | 55.059 |  |
| Constant | 363.685 | 158.001 | 2.30 | .022 | 52.445 | | 674.925 | ** |
| Mean dependent var | 455.882 | | SD dependent var | | | 192.948 | | |
| R-squared | 0.110 | | Number of obs. | | | 255 | | |
| F-test | 2.298 | | Prob > F | | | 0.007 | | |
| Akaike crit. (AIC) | 3,404.697 | | Bayesian crit. (BIC) | | | 3,454.274 | | |
| *Abbreviations: *** p<.01, ** p<.05, * p<.1*  *Legend: statistically significant results* | | | | | | | | |

**(II) Analysis "Choice of ‘no treatment’"**

**II.1 Correlation and regression analysis for posterior teeth (PT)**

**II.1.1 Correlation analysis – PT**

| **Variables** | **(1)** | **(2)** | **(3)** | **(4)** | **(5)** | **(6)** | **(7)** | **(8)** | **(9)** | **(10)** | **(11)** | **(12)** | **(13)** | **(14)** | **(15)** | **(16)** | **(17)** |
| --- | --- | --- | --- | --- | --- | --- | --- | --- | --- | --- | --- | --- | --- | --- | --- | --- | --- |
| (1) ‘No treatment’† (PT) | 1.000 |  |  |  |  |  |  |  |  |  |  |  |  |  |  |  |  |
|  |  |  |  |  |  |  |  |  |  |  |  |  |  |  |  |  |  |
| (2) Age | 0.169* | 1.000 |  |  |  |  |  |  |  |  |  |  |  |  |  |  |  |
|  | (0.001) |  |  |  |  |  |  |  |  |  |  |  |  |  |  |  |  |
| Gender | | | | | | | | | | | | | | | | | |
| (3) Female | 0.016 | -0.166* | 1.000 |  |  |  |  |  |  |  |  |  |  |  |  |  |  |
|  | (0.758) | (0.002) |  |  |  |  |  |  |  |  |  |  |  |  |  |  |  |
| (4) Male | -0.011 | 0.167* | -0.994* | 1.000 |  |  |  |  |  |  |  |  |  |  |  |  |  |
|  | (0.828) | (0.001) | (0.000) |  |  |  |  |  |  |  |  |  |  |  |  |  |  |
| (5) Other (gender) | -0.044 | -0.011 | -0.071 | -0.037 | 1.000 |  |  |  |  |  |  |  |  |  |  |  |  |
|  | (0.393) | (0.832) | (0.168) | (0.472) |  |  |  |  |  |  |  |  |  |  |  |  |  |
| (6) Income | -0.052 | -0.169* | -0.117* | 0.121* | -0.029 | 1.000 |  |  |  |  |  |  |  |  |  |  |  |
|  | (0.327) | (0.002) | (0.028) | (0.023) | (0.585) |  |  |  |  |  |  |  |  |  |  |  |  |
| Employment | | | | | | | | | | | | | | | | | |
| (7) Full time employed | -0.088 | -0.537* | -0.074 | 0.068 | 0.054 | 0.334* | 1.000 |  |  |  |  |  |  |  |  |  |  |
|  | (0.099) | (0.000) | (0.165) | (0.201) | (0.312) | (0.000) |  |  |  |  |  |  |  |  |  |  |  |
| (8) Not full time employed or retired | 0.088 | 0.537* | 0.074 | -0.068 | -0.054 | -0.334* | -1.000 | 1.000 |  |  |  |  |  |  |  |  |  |
|  | (0.099) | (0.000) | (0.165) | (0.201) | (0.312) | (0.000) | (1.000) |  |  |  |  |  |  |  |  |  |  |
| (9) Education | -0.013 | 0.015 | 0.019 | -0.026 | 0.058 | -0.232* | -0.005 | 0.005 | 1.000 |  |  |  |  |  |  |  |  |
|  | (0.805) | (0.784) | (0.725) | (0.635) | (0.287) | (0.000) | (0.931) | (0.931) |  |  |  |  |  |  |  |  |  |
| (10) Rural / urban region | -0.036 | 0.090 | -0.111* | 0.105* | 0.052 | 0.046 | -0.044 | 0.044 | -0.029 | 1.000 |  |  |  |  |  |  |  |
|  | (0.503) | (0.101) | (0.037) | (0.048) | (0.326) | (0.402) | (0.433) | (0.433) | (0.603) |  |  |  |  |  |  |  |  |
| Importance of attributes | | | | | | | | | | | | | | | | | |
| (11) Aesthetics | 0.101 | -0.054 | 0.144* | -0.144* | -0.006 | 0.041 | 0.054 | -0.054 | 0.002 | 0.083 | 1.000 |  |  |  |  |  |  |
|  | (0.051) | (0.314) | (0.005) | (0.005) | (0.914) | (0.443) | (0.313) | (0.313) | (0.971) | (0.121) |  |  |  |  |  |  |  |
| (12) Compatibility | 0.110* | 0.008 | 0.164* | -0.153* | -0.107* | 0.029 | 0.020 | -0.020 | -0.057 | 0.043 | 0.329* | 1.000 |  |  |  |  |  |
|  | (0.034) | (0.886) | (0.001) | (0.003) | (0.039) | (0.595) | (0.711) | (0.711) | (0.299) | (0.427) | (0.000) |  |  |  |  |  |  |
| (13) Duration | 0.040 | -0.098 | 0.063 | -0.067 | 0.030 | 0.093 | 0.102 | -0.102 | -0.067 | 0.000 | 0.133* | 0.382* | 1.000 |  |  |  |  |
|  | (0.438) | (0.065) | (0.222) | (0.199) | (0.567) | (0.083) | (0.058) | (0.058) | (0.222) | (1.000) | (0.010) | (0.000) |  |  |  |  |  |
| (14) Out-of-pocket payment | 0.041 | -0.009 | 0.124* | -0.120* | -0.034 | -0.243* | -0.054 | 0.054 | 0.110* | -0.062 | 0.024 | 0.035 | 0.116* | 1.000 |  |  |  |
|  | (0.425) | (0.872) | (0.017) | (0.020) | (0.509) | (0.000) | (0.318) | (0.318) | (0.044) | (0.251) | (0.645) | (0.498) | (0.025) |  |  |  |  |
| Incentive measures | | | | | | | | | | | | | | | | | |
| (15) Bonus booklet | -0.026 | 0.242* | 0.129* | -0.132* | 0.019 | -0.016 | -0.067 | 0.067 | 0.004 | -0.112* | 0.038 | 0.093 | -0.035 | 0.013 | 1.000 |  |  |
|  | (0.610) | (0.000) | (0.012) | (0.011) | (0.712) | (0.769) | (0.215) | (0.215) | (0.939) | (0.036) | (0.465) | (0.074) | (0.505) | (0.807) |  |  |  |
| (16) Supplementary insurance | 0.082 | 0.002 | 0.112* | -0.109* | -0.035 | 0.014 | -0.019 | 0.019 | 0.073 | 0.044 | 0.127* | 0.086 | 0.052 | 0.008 | 0.161* | 1.000 |  |
|  | (0.114) | (0.970) | (0.030) | (0.036) | (0.502) | (0.799) | (0.729) | (0.729) | (0.184) | (0.408) | (0.015) | (0.101) | (0.323) | (0.873) | (0.002) |  |  |
| (17) Combination of bonus booklet & suppl. insurance | 0.082 | 0.028 | 0.113* | -0.109* | -0.033 | 0.012 | -0.011 | 0.011 | 0.068 | 0.026 | 0.119* | 0.112* | 0.037 | 0.005 | 0.240* | 0.969* | 1.000 |
|  | (0.109) | (0.596) | (0.028) | (0.033) | (0.521) | (0.816) | (0.837) | (0.837) | (0.213) | (0.631) | (0.022) | (0.030) | (0.474) | (0.920) | (0.000) | (0.000) |  |
| *Abbreviations: † participant has decided against a treatment in the choice-set decision between treatment A, B, or no treatment (opt-out); *** p<0.01, ** p<0.05, * p<0.1*  *Legend: statistically significant results regarding dependent variable, assumed multicollinearity (one variable to be excluded)* | | | | | | | | | | | | | | | | | |

**II.1.2 Regression analysis – PT**

| **Variables** | **Coef.** | **Std. Err.** | **t-value** | **p-value** | **[95% Conf. interval]** | | | **Sig.** |
| --- | --- | --- | --- | --- | --- | --- | --- | --- |
| Age | .211 | .069 | 3.06 | .002 | .075 | | .346 | *** |
| Gender | | | | | | | | |
| Female | .411 | .357 | 1.15 | .25 | -.291 | | 1.114 |  |
| Other (gender) | -.516 | 2.439 | -0.21 | .833 | -5.322 | | 4.289 |  |
| Income | .049 | .103 | 0.48 | .635 | -.154 | | .252 |  |
| Employment | | | | | | | | |
| Full time employed | -.098 | .385 | -0.25 | .799 | -.856 | | .66 |  |
| Education | .024 | .139 | 0.17 | .861 | -.249 | | .298 |  |
| Rural / urban region | -.244 | .114 | -2.15 | .033 | -.468 | | -.02 | ** |
| Importance of attributes | | | | | | | | |
| Aesthetics | .228 | .152 | 1.49 | .137 | -.073 | | .528 |  |
| Compatibility | .213 | .265 | 0.80 | .424 | -.31 | | .735 |  |
| Duration | -.048 | .329 | -0.15 | .884 | -.696 | | .6 |  |
| Out-of-pocket payment | .103 | .169 | 0.61 | .542 | -.23 | | .437 |  |
| Incentive measures | | | | | | | | |
| Bonus booklet | -1.208 | .495 | -2.44 | .015 | -2.182 | | -.233 | ** |
| Supplementary insurance | .707 | .337 | 2.10 | .037 | .043 | | 1.371 | ** |
| Constant | -.175 | 1.858 | -0.09 | .925 | -3.835 | | 3.485 |  |
| Mean dependent var. | 2.060 | | SD dependent var. | | | 2.435 | | |
| R-squared | 0.108 | | Number of obs. | | | 252 | | |
| F-test | 2.226 | | Prob > F | | | 0.009 | | |
| Akaike crit. (AIC) | 1,161.737 | | Bayesian crit. (BIC) | | | 1,211.149 | | |
| *Abbreviations: *** p<.01, ** p<.05, * p<.1*  *Legend: statistically significant results* | | | | | | | | |

**II.2 Correlation and regression analysis for anterior teeth (AT)**

**II.2.1 Correlation analysis – AT**

| **Variables** | **(1)** | **(2)** | **(3)** | **(4)** | **(5)** | **(6)** | **(7)** | **(8)** | **(9)** | **(10)** | **(11)** | **(12)** | **(13)** | **(14)** | **(15)** | **(16)** | **(17)** |
| --- | --- | --- | --- | --- | --- | --- | --- | --- | --- | --- | --- | --- | --- | --- | --- | --- | --- |
| (1) ‘No treatment’† (AT) | 1.000 |  |  |  |  |  |  |  |  |  |  |  |  |  |  |  |  |
|  |  |  |  |  |  |  |  |  |  |  |  |  |  |  |  |  |  |
| (2) Age | 0.163* | 1.000 |  |  |  |  |  |  |  |  |  |  |  |  |  |  |  |
|  | (0.002) |  |  |  |  |  |  |  |  |  |  |  |  |  |  |  |  |
| Gender | | | | | | | | | | | | | | | | | |
| (3) Female | 0.061 | -0.166* | 1.000 |  |  |  |  |  |  |  |  |  |  |  |  |  |  |
|  | (0.234) | (0.002) |  |  |  |  |  |  |  |  |  |  |  |  |  |  |  |
| (4) Male | -0.055 | 0.167* | -0.994* | 1.000 |  |  |  |  |  |  |  |  |  |  |  |  |  |
|  | (0.285) | (0.001) | (0.000) |  |  |  |  |  |  |  |  |  |  |  |  |  |  |
| (5) Other (gender) | -0.058 | -0.011 | -0.071 | -0.037 | 1.000 |  |  |  |  |  |  |  |  |  |  |  |  |
|  | (0.258) | (0.832) | (0.168) | (0.472) |  |  |  |  |  |  |  |  |  |  |  |  |  |
| (6) Income | -0.049 | -0.169* | -0.117* | 0.121* | -0.029 | 1.000 |  |  |  |  |  |  |  |  |  |  |  |
|  | (0.362) | (0.002) | (0.028) | (0.023) | (0.585) |  |  |  |  |  |  |  |  |  |  |  |  |
| Employment | | | | | | | | | | | | | | | | | |
| (7) Full time employed | -0.121* | -0.537* | -0.074 | 0.068 | 0.054 | 0.334* | 1.000 |  |  |  |  |  |  |  |  |  |  |
|  | (0.023) | (0.000) | (0.165) | (0.201) | (0.312) | (0.000) |  |  |  |  |  |  |  |  |  |  |  |
| (8) Not full time employed or retired | 0.121* | 0.537* | 0.074 | -0.068 | -0.054 | -0.334* | -1.000 | 1.000 |  |  |  |  |  |  |  |  |  |
|  | (0.023) | (0.000) | (0.165) | (0.201) | (0.312) | (0.000) | (1.000) |  |  |  |  |  |  |  |  |  |  |
| (9) Education | -0.066 | 0.015 | 0.019 | -0.026 | 0.058 | -0.232* | -0.005 | 0.005 | 1.000 |  |  |  |  |  |  |  |  |
|  | (0.222) | (0.784) | (0.725) | (0.635) | (0.287) | (0.000) | (0.931) | (0.931) |  |  |  |  |  |  |  |  |  |
| (10) Rural / urban region | -0.041 | 0.090 | -0.111* | 0.105* | 0.052 | 0.046 | -0.044 | 0.044 | -0.029 | 1.000 |  |  |  |  |  |  |  |
|  | (0.440) | (0.101) | (0.037) | (0.048) | (0.326) | (0.402) | (0.433) | (0.433) | (0.603) |  |  |  |  |  |  |  |  |
| Importance of attributes | | | | | | | | | | | | | | | | | |
| (11) Aesthetics | 0.018 | -0.180* | 0.263* | -0.267* | 0.025 | 0.129* | 0.123* | -0.123* | -0.067 | -0.066 | 1.000 |  |  |  |  |  |  |
|  | (0.723) | (0.001) | (0.000) | (0.000) | (0.631) | (0.016) | (0.021) | (0.021) | (0.216) | (0.216) |  |  |  |  |  |  |  |
| (12) Compatibility | 0.109* | 0.038 | 0.213* | -0.217* | 0.039 | -0.027 | -0.044 | 0.044 | 0.016 | -0.056 | 0.288* | 1.000 |  |  |  |  |  |
|  | (0.035) | (0.474) | (0.000) | (0.000) | (0.455) | (0.608) | (0.418) | (0.418) | (0.770) | (0.290) | (0.000) |  |  |  |  |  |  |
| (13) Duration | 0.083 | 0.027 | 0.211* | -0.215* | 0.032 | 0.010 | -0.019 | 0.019 | -0.098 | -0.011 | 0.276* | 0.477* | 1.000 |  |  |  |  |
|  | (0.106) | (0.612) | (0.000) | (0.000) | (0.530) | (0.859) | (0.728) | (0.728) | (0.071) | (0.837) | (0.000) | (0.000) |  |  |  |  |  |
| (14) Out-of-pocket payment | 0.058 | -0.027 | 0.138* | -0.145* | 0.066 | -0.304* | -0.012 | 0.012 | 0.179* | 0.043 | 0.135* | 0.234* | 0.236* | 1.000 |  |  |  |
|  | (0.264) | (0.618) | (0.007) | (0.005) | (0.203) | (0.000) | (0.823) | (0.823) | (0.001) | (0.417) | (0.009) | (0.000) | (0.000) |  |  |  |  |
| Incentive measures | | | | | | | | | | | | | | | | | |
| (15) Bonus booklet | -0.011 | 0.242* | 0.129* | -0.132* | 0.019 | -0.016 | -0.067 | 0.067 | 0.004 | -0.112* | 0.075 | 0.139* | 0.027 | -0.037 | 1.000 |  |  |
|  | (0.829) | (0.000) | (0.012) | (0.011) | (0.712) | (0.769) | (0.215) | (0.215) | (0.939) | (0.036) | (0.151) | (0.007) | (0.600) | (0.481) |  |  |  |
| (16) Supplementary insurance | 0.103* | 0.002 | 0.112* | -0.109* | -0.035 | 0.014 | -0.019 | 0.019 | 0.073 | 0.044 | 0.145* | 0.173* | 0.161* | 0.034 | 0.161* | 1.000 |  |
|  | (0.047) | (0.970) | (0.030) | (0.036) | (0.502) | (0.799) | (0.729) | (0.729) | (0.184) | (0.408) | (0.005) | (0.001) | (0.002) | (0.516) | (0.002) |  |  |
| (17) Combination of bonus booklet & suppl. insurance | 0.096 | 0.028 | 0.113* | -0.109* | -0.033 | 0.012 | -0.011 | 0.011 | 0.068 | 0.026 | 0.131* | 0.200* | 0.141* | 0.025 | 0.240* | 0.969* | 1.000 |
|  | (0.062) | (0.596) | (0.028) | (0.033) | (0.521) | (0.816) | (0.837) | (0.837) | (0.213) | (0.631) | (0.011) | (0.000) | (0.006) | (0.623) | (0.000) | (0.000) |  |
| *Abbreviations: † participant has decided against a treatment in the choice-set decision between treatment A, B, or no treatment (opt-out); *** p<0.01, ** p<0.05, * p<0.1*  *Legend: statistically significant results regarding dependent variable, assumed multicollinearity (one variable to be excluded)* | | | | | | | | | | | | | | | | | |

**II.2.2 Regression analysis – AT**

| **Variables** | **Coef.** | **Std. Err.** | **t-value** | **p-value** | **[95% Conf. interval]** | | | **Sig.** |
| --- | --- | --- | --- | --- | --- | --- | --- | --- |
| Age | .21 | .075 | 2.80 | .005 | .062 | | .357 | *** |
| Gender | | | | | | | | |
| Female | .81 | .394 | 2.05 | .041 | .033 | | 1.586 | ** |
| Other (gender) | -1.575 | 2.641 | -0.60 | .551 | -6.776 | | 3.627 |  |
| Income | .125 | .115 | 1.09 | .278 | -.102 | | .352 |  |
| Employment | | | | | | | | |
| Full time employed | -.196 | .414 | -0.47 | .637 | -1.011 | | .62 |  |
| Education | -.132 | .153 | -0.86 | .39 | -.433 | | .17 |  |
| Rural / urban region | -.254 | .123 | -2.07 | .04 | -.496 | | -.012 | ** |
| Importance of attributes | | | | | | | | |
| Aesthetics | -.001 | .302 | -0.00 | .998 | -.596 | | .595 |  |
| Compatibility | .281 | .292 | 0.96 | .338 | -.296 | | .857 |  |
| Duration | -.046 | .373 | -0.12 | .901 | -.782 | | .689 |  |
| Out-of-pocket payment | .163 | .182 | 0.90 | .371 | -.195 | | .52 |  |
| Incentive measures | | | | | | | | |
| Bonus booklet | -1.07 | .545 | -1.96 | .051 | -2.143 | | .004 | * |
| Supplementary insurance | .941 | .368 | 2.56 | .011 | .216 | | 1.666 | ** |
| Constant | .387 | 2.185 | 0.18 | .859 | -3.916 | | 4.691 |  |
| Mean dependent var. | 3.012 | | SD dependent var. | | | 2.678 | | |
| R-squared | 0.117 | | Number of obs. | | | 255 | | |
| F-test | 2.448 | | Prob > F | | | 0.004 | | |
| Akaike crit. (AIC) | 1,221.326 | | Bayesian crit. (BIC) | | | 1,270.904 | | |
| *Abbreviations: *** p<.01, ** p<.05, * p<.1*  *Legend: statistically significant results* | | | | | | | | |

**(III) Analysis "Choice of ‘SHI+ treatment’"**

**III.1 Correlation and regression analysis for posterior teeth (PT)**

**III1.1 Correlation analysis – PT**

| **Variables** | **(1)** | **(2)** | **(3)** | **(4)** | **(5)** | **(6)** | **(7)** | **(8)** | **(9)** | **(10)** | **(11)** | **(12)** | **(13)** | **(14)** | **(15)** | **(16)** | **(17)** |
| --- | --- | --- | --- | --- | --- | --- | --- | --- | --- | --- | --- | --- | --- | --- | --- | --- | --- |
| (1) ‘SHI+ treatment’† (PT) | 1.000 |  |  |  |  |  |  |  |  |  |  |  |  |  |  |  |  |
|  |  |  |  |  |  |  |  |  |  |  |  |  |  |  |  |  |  |
| (2) Age | -0.083 | 1.000 |  |  |  |  |  |  |  |  |  |  |  |  |  |  |  |
|  | (0.115) |  |  |  |  |  |  |  |  |  |  |  |  |  |  |  |  |
| Gender | | | | | | | | | | | | | | | | | |
| (3) Female | 0.001 | -0.166* | 1.000 |  |  |  |  |  |  |  |  |  |  |  |  |  |  |
|  | (0.977) | (0.002) |  |  |  |  |  |  |  |  |  |  |  |  |  |  |  |
| (4) Male | -0.009 | 0.167* | -0.994* | 1.000 |  |  |  |  |  |  |  |  |  |  |  |  |  |
|  | (0.863) | (0.001) | (0.000) |  |  |  |  |  |  |  |  |  |  |  |  |  |  |
| (5) Other (gender) | 0.069 | -0.011 | -0.071 | -0.037 | 1.000 |  |  |  |  |  |  |  |  |  |  |  |  |
|  | (0.182) | (0.832) | (0.168) | (0.472) |  |  |  |  |  |  |  |  |  |  |  |  |  |
| (6) Income | 0.122* | -0.169* | -0.117* | 0.121* | -0.029 | 1.000 |  |  |  |  |  |  |  |  |  |  |  |
|  | (0.021) | (0.002) | (0.028) | (0.023) | (0.585) |  |  |  |  |  |  |  |  |  |  |  |  |
| Employment | | | | | | | | | | | | | | | | | |
| (7) Full time employed | 0.083 | -0.537* | -0.074 | 0.068 | 0.054 | 0.334* | 1.000 |  |  |  |  |  |  |  |  |  |  |
|  | (0.122) | (0.000) | (0.165) | (0.201) | (0.312) | (0.000) |  |  |  |  |  |  |  |  |  |  |  |
| (8) Not full time employed or retired | -0.083 | 0.537* | 0.074 | -0.068 | -0.054 | -0.334* | -1.000 | 1.000 |  |  |  |  |  |  |  |  |  |
|  | (0.122) | (0.000) | (0.165) | (0.201) | (0.312) | (0.000) | (1.000) |  |  |  |  |  |  |  |  |  |  |
| (9) Education | -0.073 | 0.015 | 0.019 | -0.026 | 0.058 | -0.232* | -0.005 | 0.005 | 1.000 |  |  |  |  |  |  |  |  |
|  | (0.178) | (0.784) | (0.725) | (0.635) | (0.287) | (0.000) | (0.931) | (0.931) |  |  |  |  |  |  |  |  |  |
| (10) Rural / urban region | -0.020 | 0.090 | -0.111* | 0.105* | 0.052 | 0.046 | -0.044 | 0.044 | -0.029 | 1.000 |  |  |  |  |  |  |  |
|  | (0.708) | (0.101) | (0.037) | (0.048) | (0.326) | (0.402) | (0.433) | (0.433) | (0.603) |  |  |  |  |  |  |  |  |
| Importance of attributes | | | | | | | | | | | | | | | | | |
| (11) Aesthetics | 0.168* | -0.054 | 0.144* | -0.144* | -0.006 | 0.041 | 0.054 | -0.054 | 0.002 | 0.083 | 1.000 |  |  |  |  |  |  |
|  | (0.001) | (0.314) | (0.005) | (0.005) | (0.914) | (0.443) | (0.313) | (0.313) | (0.971) | (0.121) |  |  |  |  |  |  |  |
| (12) Compatibility | -0.007 | 0.008 | 0.164* | -0.153* | -0.107* | 0.029 | 0.020 | -0.020 | -0.057 | 0.043 | 0.329* | 1.000 |  |  |  |  |  |
|  | (0.897) | (0.886) | (0.001) | (0.003) | (0.039) | (0.595) | (0.711) | (0.711) | (0.299) | (0.427) | (0.000) |  |  |  |  |  |  |
| (13) Duration | 0.014 | -0.098 | 0.063 | -0.067 | 0.030 | 0.093 | 0.102 | -0.102 | -0.067 | 0.000 | 0.133* | 0.382* | 1.000 |  |  |  |  |
|  | (0.788) | (0.065) | (0.222) | (0.199) | (0.567) | (0.083) | (0.058) | (0.058) | (0.222) | (1.000) | (0.010) | (0.000) |  |  |  |  |  |
| (14) Out-of-pocket payment | -0.139* | -0.009 | 0.124* | -0.120* | -0.034 | -0.243* | -0.054 | 0.054 | 0.110* | -0.062 | 0.024 | 0.035 | 0.116* | 1.000 |  |  |  |
|  | (0.007) | (0.872) | (0.017) | (0.020) | (0.509) | (0.000) | (0.318) | (0.318) | (0.044) | (0.251) | (0.645) | (0.498) | (0.025) |  |  |  |  |
| Incentive measures | | | | | | | | | | | | | | | | | |
| (15) Bonus booklet | 0.111* | 0.242* | 0.129* | -0.132* | 0.019 | -0.016 | -0.067 | 0.067 | 0.004 | -0.112* | 0.038 | 0.093 | -0.035 | 0.013 | 1.000 |  |  |
|  | (0.032) | (0.000) | (0.012) | (0.011) | (0.712) | (0.769) | (0.215) | (0.215) | (0.939) | (0.036) | (0.465) | (0.074) | (0.505) | (0.807) |  |  |  |
| (16) Supplementary insurance | 0.067 | 0.002 | 0.112* | -0.109* | -0.035 | 0.014 | -0.019 | 0.019 | 0.073 | 0.044 | 0.127* | 0.086 | 0.052 | 0.008 | 0.161* | 1.000 |  |
|  | (0.196) | (0.970) | (0.030) | (0.036) | (0.502) | (0.799) | (0.729) | (0.729) | (0.184) | (0.408) | (0.015) | (0.101) | (0.323) | (0.873) | (0.002) |  |  |
| (17) Combination of bonus booklet & suppl. insurance | 0.062 | 0.028 | 0.113* | -0.109* | -0.033 | 0.012 | -0.011 | 0.011 | 0.068 | 0.026 | 0.119* | 0.112* | 0.037 | 0.005 | 0.240* | 0.969* | 1.000 |
|  | (0.227) | (0.596) | (0.028) | (0.033) | (0.521) | (0.816) | (0.837) | (0.837) | (0.213) | (0.631) | (0.022) | (0.030) | (0.474) | (0.920) | (0.000) | (0.000) |  |
| *Abbreviations: † participant has decided for a treatment presenting attribute levels beyond statutory health insurance (SHI) standard care; *** p<0.01, ** p<0.05, * p<0.1*  *Legend: statistically significant results regarding dependent variable, assumed multicollinearity (one variable to be excluded)* | | | | | | | | | | | | | | | | | |

**III.1.2 Regression analysis – PT**

| **Variables** | **Coef.** | **Std. Err.** | **t-value** | **p-value** | **[95% Conf. interval]** | | | **Sig.** |
| --- | --- | --- | --- | --- | --- | --- | --- | --- |
| Age | -.064 | .04 | -1.60 | .11 | -.143 | | .015 |  |
| Gender | | | | | | | | |
| Female | -.255 | .207 | -1.23 | .219 | -.663 | | .153 |  |
| Other (gender) | 1.137 | 1.416 | 0.80 | .423 | -1.654 | | 3.927 |  |
| Income | -.014 | .06 | -0.24 | .814 | -.132 | | .104 |  |
| Employment | | | | | | | | |
| Full time employed | .079 | .223 | 0.35 | .725 | -.362 | | .519 |  |
| Education | -.083 | .081 | -1.03 | .303 | -.242 | | .076 |  |
| Rural / urban region | .055 | .066 | 0.84 | .404 | -.075 | | .185 |  |
| Importance of attributes | | | | | | | | |
| Aesthetics | .255 | .089 | 2.88 | .004 | .08 | | .429 | *** |
| Compatibility | -.205 | .154 | -1.33 | .185 | -.509 | | .099 |  |
| Duration | .092 | .191 | 0.48 | .631 | -.284 | | .468 |  |
| Out-of-pocket payment | -.214 | .098 | -2.18 | .031 | -.407 | | -.02 | ** |
| Incentive measures | | | | | | | | |
| Bonus booklet | .957 | .287 | 3.33 | .001 | .391 | | 1.523 | *** |
| Supplementary insurance | -.113 | .196 | -0.58 | .566 | -.498 | | .273 |  |
| Constant | 2.345 | 1.079 | 2.17 | .031 | .22 | | 4.47 | ** |
| Mean dependent var. | 2.036 | | SD dependent var. | | | 1.421 | | |
| R-squared | 0.117 | | Number of obs. | | | 252 | | |
| F-test | 2.431 | | Prob > F | | | 0.004 | | |
| Akaike crit. (AIC) | 887.730 | | Bayesian crit. (BIC) | | | 937.142 | | |
| *Abbreviations: *** p<.01, ** p<.05, * p<.1*  *Legend: statistically significant results* | | | | | | | | |

**III.2 Correlation and regression analysis for anterior teeth (AT)**

**III.2.1 Correlation analysis – AT**

| **Variables** | **(1)** | **(2)** | **(3)** | **(4)** | **(5)** | **(6)** | **(7)** | **(8)** | **(9)** | **(10)** | **(11)** | **(12)** | **(13)** | **(14)** | **(15)** | **(16)** | **(17)** |
| --- | --- | --- | --- | --- | --- | --- | --- | --- | --- | --- | --- | --- | --- | --- | --- | --- | --- |
| (1) ‘SHI+ treatment’† (AT) | 1.000 |  |  |  |  |  |  |  |  |  |  |  |  |  |  |  |  |
|  |  |  |  |  |  |  |  |  |  |  |  |  |  |  |  |  |  |
| (2) Age | -0.121* | 1.000 |  |  |  |  |  |  |  |  |  |  |  |  |  |  |  |
|  | (0.022) |  |  |  |  |  |  |  |  |  |  |  |  |  |  |  |  |
| Gender | | | | | | | | | | | | | | | | | |
| (3) Female | -0.061 | -0.166* | 1.000 |  |  |  |  |  |  |  |  |  |  |  |  |  |  |
|  | (0.239) | (0.002) |  |  |  |  |  |  |  |  |  |  |  |  |  |  |  |
| (4) Male | 0.054 | 0.167* | -0.994* | 1.000 |  |  |  |  |  |  |  |  |  |  |  |  |  |
|  | (0.293) | (0.001) | (0.000) |  |  |  |  |  |  |  |  |  |  |  |  |  |  |
| (5) Other (gender) | 0.061 | -0.011 | -0.071 | -0.037 | 1.000 |  |  |  |  |  |  |  |  |  |  |  |  |
|  | (0.237) | (0.832) | (0.168) | (0.472) |  |  |  |  |  |  |  |  |  |  |  |  |  |
| (6) Income | 0.103 | -0.169* | -0.117* | 0.121* | -0.029 | 1.000 |  |  |  |  |  |  |  |  |  |  |  |
|  | (0.053) | (0.002) | (0.028) | (0.023) | (0.585) |  |  |  |  |  |  |  |  |  |  |  |  |
| Employment | | | | | | | | | | | | | | | | | |
| (7) Full time employed | 0.134* | -0.537* | -0.074 | 0.068 | 0.054 | 0.334* | 1.000 |  |  |  |  |  |  |  |  |  |  |
|  | (0.012) | (0.000) | (0.165) | (0.201) | (0.312) | (0.000) |  |  |  |  |  |  |  |  |  |  |  |
| (8) Not full time employed or retired | -0.134* | 0.537* | 0.074 | -0.068 | -0.054 | -0.334* | -1.000 | 1.000 |  |  |  |  |  |  |  |  |  |
|  | (0.012) | (0.000) | (0.165) | (0.201) | (0.312) | (0.000) | (1.000) |  |  |  |  |  |  |  |  |  |  |
| (9) Education | 0.022 | 0.015 | 0.019 | -0.026 | 0.058 | -0.232* | -0.005 | 0.005 | 1.000 |  |  |  |  |  |  |  |  |
|  | (0.681) | (0.784) | (0.725) | (0.635) | (0.287) | (0.000) | (0.931) | (0.931) |  |  |  |  |  |  |  |  |  |
| (10) Rural / urban region | 0.057 | 0.090 | -0.111* | 0.105* | 0.052 | 0.046 | -0.044 | 0.044 | -0.029 | 1.000 |  |  |  |  |  |  |  |
|  | (0.287) | (0.101) | (0.037) | (0.048) | (0.326) | (0.402) | (0.433) | (0.433) | (0.603) |  |  |  |  |  |  |  |  |
| Importance of attributes | | | | | | | | | | | | | | | | | |
| (11) Aesthetics | 0.030 | -0.180* | 0.263* | -0.267* | 0.025 | 0.129* | 0.123* | -0.123* | -0.067 | -0.066 | 1.000 |  |  |  |  |  |  |
|  | (0.560) | (0.001) | (0.000) | (0.000) | (0.631) | (0.016) | (0.021) | (0.021) | (0.216) | (0.216) |  |  |  |  |  |  |  |
| (12) Compatibility | -0.067 | 0.038 | 0.213* | -0.217* | 0.039 | -0.027 | -0.044 | 0.044 | 0.016 | -0.056 | 0.288* | 1.000 |  |  |  |  |  |
|  | (0.193) | (0.474) | (0.000) | (0.000) | (0.455) | (0.608) | (0.418) | (0.418) | (0.770) | (0.290) | (0.000) |  |  |  |  |  |  |
| (13) Duration | -0.018 | 0.027 | 0.211* | -0.215* | 0.032 | 0.010 | -0.019 | 0.019 | -0.098 | -0.011 | 0.276* | 0.477* | 1.000 |  |  |  |  |
|  | (0.733) | (0.612) | (0.000) | (0.000) | (0.530) | (0.859) | (0.728) | (0.728) | (0.071) | (0.837) | (0.000) | (0.000) |  |  |  |  |  |
| (14) Out-of-pocket payment | -0.094 | -0.027 | 0.138* | -0.145* | 0.066 | -0.304* | -0.012 | 0.012 | 0.179* | 0.043 | 0.135* | 0.234* | 0.236* | 1.000 |  |  |  |
|  | (0.067) | (0.618) | (0.007) | (0.005) | (0.203) | (0.000) | (0.823) | (0.823) | (0.001) | (0.417) | (0.009) | (0.000) | (0.000) |  |  |  |  |
| Incentive measures | | | | | | | | | | | | | | | | | |
| (15) Bonus booklet | 0.021 | 0.242* | 0.129* | -0.132* | 0.019 | -0.016 | -0.067 | 0.067 | 0.004 | -0.112* | 0.075 | 0.139* | 0.027 | -0.037 | 1.000 |  |  |
|  | (0.687) | (0.000) | (0.012) | (0.011) | (0.712) | (0.769) | (0.215) | (0.215) | (0.939) | (0.036) | (0.151) | (0.007) | (0.600) | (0.481) |  |  |  |
| (16) Supplementary insurance | -0.004 | 0.002 | 0.112* | -0.109* | -0.035 | 0.014 | -0.019 | 0.019 | 0.073 | 0.044 | 0.145* | 0.173* | 0.161* | 0.034 | 0.161* | 1.000 |  |
|  | (0.942) | (0.970) | (0.030) | (0.036) | (0.502) | (0.799) | (0.729) | (0.729) | (0.184) | (0.408) | (0.005) | (0.001) | (0.002) | (0.516) | (0.002) |  |  |
| (17) Combination of bonus booklet & suppl. insurance | 0.002 | 0.028 | 0.113* | -0.109* | -0.033 | 0.012 | -0.011 | 0.011 | 0.068 | 0.026 | 0.131* | 0.200* | 0.141* | 0.025 | 0.240* | 0.969* | 1.000 |
|  | (0.973) | (0.596) | (0.028) | (0.033) | (0.521) | (0.816) | (0.837) | (0.837) | (0.213) | (0.631) | (0.011) | (0.000) | (0.006) | (0.623) | (0.000) | (0.000) |  |
| *Abbreviations: † participant has decided for a treatment presenting attribute levels beyond statutory health insurance (SHI) standard care; *** p<0.01, ** p<0.05, * p<0.1*  *Legend: statistically significant results regarding dependent variable, assumed multicollinearity (one variable to be excluded)* | | | | | | | | | | | | | | | | | |

**III.2.2 Regression analysis – AT**

| **Variables** | **Coef.** | **Std. Err.** | **t-value** | **p-value** | **[95% Conf. Interval]** | | | **Sig.** |
| --- | --- | --- | --- | --- | --- | --- | --- | --- |
| Age | -.079 | .044 | -1.82 | .07 | -.165 | | .007 | * |
| Gender | | | | | | | | |
| Female | -.513 | .23 | -2.23 | .026 | -.965 | | -.06 | ** |
| Other (gender) | 1.223 | 1.538 | 0.80 | .427 | -1.805 | | 4.252 |  |
| Income | -.034 | .067 | -0.51 | .609 | -.166 | | .098 |  |
| Employment | | | | | | | | |
| Full time employed | .172 | .241 | 0.71 | .477 | -.303 | | .647 |  |
| Education | .074 | .089 | 0.83 | .408 | -.102 | | .25 |  |
| Rural / urban region | .169 | .072 | 2.36 | .019 | .028 | | .31 | ** |
| Importance of attributes | | | | | | | | |
| Aesthetics | .114 | .176 | 0.65 | .517 | -.232 | | .461 |  |
| Compatibility | -.189 | .17 | -1.11 | .269 | -.524 | | .147 |  |
| Duration | .309 | .217 | 1.42 | .157 | -.119 | | .737 |  |
| Out-of-pocket payment | -.207 | .106 | -1.96 | .051 | -.416 | | .001 | * |
| Incentive measures | | | | | | | | |
| Bonus booklet | .736 | .317 | 2.32 | .021 | .111 | | 1.361 | ** |
| Supplementary insurance | -.254 | .214 | -1.19 | .237 | -.676 | | .168 |  |
| Constant | 1.481 | 1.272 | 1.16 | .245 | -1.025 | | 3.987 |  |
| Mean dependent var. | 2.039 | | SD dependent var. | | | 1.549 | | |
| R-squared | 0.105 | | Number of obs. | | | 255 | | |
| F-test | 2.180 | | Prob > F | | | 0.011 | | |
| Akaike crit. (AIC) | 945.554 | | Bayesian crit. (BIC) | | | 995.132 | | |
| *Abbreviations: *** p<.01, ** p<.05, * p<.1*  *Legend: statistically significant results* | | | | | | | | |
